# Incidence and risk of hypertension and proteinuria in cancer patients treated with lenvatinib: a systematic review and meta-analysis

**DOI:** 10.1093/oncolo/oyaf203

**Published:** 2025-07-09

**Authors:** Yuma Shibutani, Atsuko Suzuki, Takuro Imaoka, Kazuko Tajiri

**Affiliations:** Department of Pharmacy, National Cancer Center Hospital East, Kashiwa, Chiba 277-8577, Japan; Department of Clinical Laboratories, National Cancer Center Hospital East, Kashiwa, Chiba 277-8577, Japan; Department of Cardiology, National Cancer Center Hospital East, Kashiwa, Chiba 277-8577, Japan; Department of Cardiology, National Cancer Center Hospital East, Kashiwa, Chiba 277-8577, Japan; Tsukuba Life Science Innovation Program (T-LSI), School of Integrative and Global Majors (SIGMA), University of Tsukuba, Tsukuba, Ibaraki 305-8575, Japan

## Abstract

**Background:**

Published data regarding the overall risks and incidence of hypertension and proteinuria associated with lenvatinib remain unclear.

**Methods:**

We performed a systematic review and meta-analysis to quantify the precise risks and incidence of lenvatinib-associated hypertension and proteinuria. We systematically searched PubMed, Cochrane Library, and Web of Science databases for studies on the incidence of hypertension and proteinuria, which were published until April 24, 2023.

**Results:**

A total of 10 443 patients were included in the 64 studies identified from the literature. The incidence of all-grade and grade ≥3 hypertension was 50% (95% confidence interval [CI]: 43%-58%) and 14% (95% CI, 10%-18%) for patients treated with lenvatinib. The incidence of all-grade and grade ≥3 proteinuria was 32% (95% CI, 26%-38%) and 6% (95% CI, 4%-7%), respectively. Compared with controls, lenvatinib significantly increased the risk of all-grade hypertension (odds ratio [OR]: 2.4, 95% CI, 1.4-4.1), grade ≥3 hypertension (OR: 2.7, 95% CI, 1.2-6.0), all-grade proteinuria (OR: 3.5, 95% CI, 1.9-6.6), and grade ≥3 proteinuria (OR: 2.85, 95% CI, 1.5-5.3). Compared to low-dose lenvatinib (≤12 mg/day), high-dose lenvatinib (≥20 mg/day) treatment significantly increased the risks of all-grade hypertension (OR: 4.7, 95% CI, 4.2-5.2), grade ≥3 hypertension (OR: 7.7, 95% CI, 6.8-8.7), all-grade proteinuria (OR: 1.8, 95% CI, 1.6-2.0), and grade ≥3 proteinuria (OR: 1.7, 95% CI, 1.5-2.1).

**Conclusion:**

Our review revealed that lenvatinib treatment increases the risks of hypertension and proteinuria, particularly with high-dose lenvatinib.

## Graphical abstract

**Graphical Abstract:**
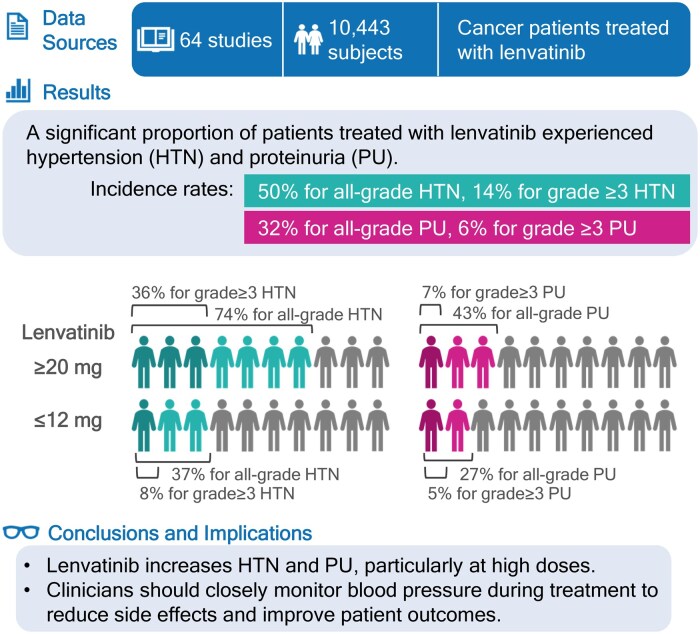

Implications for Practice Lenvatinib frequently causes hypertension and proteinuria, with severe cases leading to cardiovascular damage and renal failure. Our meta-analysis found that lenvatinib-treated patients showed high incidences of hypertension (50% for all-grade, 14% for grade ≥3) and proteinuria (30% for all-grade, 6% for grade ≥3) relative to controls. Notably, high-dose lenvatinib treatment presented greater risks than low-dose treatment. These results underscore the necessity for careful blood pressure monitoring, especially at high doses, to help prevent severe complications and enhance patient outcomes during lenvatinib therapy.

## Introduction

Tumor cells induce angiogenesis through the vascular endothelial growth factor (VEGF), which leads to tumor growth and metastasis.^1^ Therefore, in recent years, greater emphasis has been placed on therapies targeting VEGF; many molecular-targeted agents have been used, such as monoclonal antibodies against VEGF and VEGF receptor (VEGFR), as well as VEGFR-tyrosine kinase inhibitors (TKIs).^2^^,^^3^ Lenvatinib is one of the VEGFR-TKIs that demonstrates strong inhibitory activity against VEGFR2. It is indicated for the treatment of various cancers, including thyroid cancer, hepatocellular carcinoma (HCC), thymic carcinoma, and endometrial cancer.^3–7^

Lenvatinib is a multikinase inhibitor that targets VEGFRs and other angiogenic and oncogenic receptor tyrosine kinases.^6–8^ The most common adverse effects of lenvatinib include hypertension and proteinuria, which are the primary factors responsible for dose interruption or reduction.^9^ Studies have shown that lenvatinib is associated with a higher risk of developing hypertension and proteinuria than other VEGFR-TKIs.^10–12^ High-grade (grade ≥3) hypertension and proteinuria, especially hypertensive crisis and nephrotic syndrome, may cause significant cardiovascular damage and renal failure.^13–16^ These life-threatening consequences can lead to treatment interruption or discontinuation, consequently worsening cancer outcomes.^17^

The incidence of grade ≥3 hypertension in patients treated with lenvatinib varies considerably, ranging from 0% in an HCC study to 74% in a thyroid cancer study.^18^^,^^19^ A similar variation exists for the incidence of high-grade proteinuria, with reports ranging from 0% in an HCC study to 33% in a thyroid cancer study.^20^^,^^21^ Owing to the limited number of patients in each study, the overall risk magnitude of developing hypertension and proteinuria in patients treated with lenvatinib remains unclear. Moreover, while hypertension and proteinuria are considered dose-limiting toxicities of lenvatinib, no reports have definitively verified this, and the frequency of these adverse effects across different therapeutic doses remains unknown. Therefore, we performed a systematic review and meta-analysis to estimate the overall risks and incidence of hypertension and proteinuria associated with lenvatinib, with a particular focus on the dosage.

## Methods

The study followed the Preferred Reporting Items for Systematic Reviews and Meta-Analyses (PRISMA) guidelines^22^ (Tables S1 and S2).

### Search strategy

A search for published studies indexed in PubMed, Cochrane Library, and Web of Science databases from inception of the study to April 24, 2023 was conducted using the following terms: (“lenvatinib” OR “lenvima” OR “E-7080” OR “E 7080” OR “E7080”) AND (“cancer” OR “carcinoma” OR “neoplasm s” OR “neoplasms” OR “neoplasm” OR “tumoral” OR “tumour” OR “tumor” OR “tumors” OR “tumoural” OR “tumourous” OR “malignancy” OR “oncology”).

### Study selection

Studies that met the following criteria were selected for analysis: (1) prospective and retrospective studies that investigated the efficacy and safety of lenvatinib as monotherapy, and (2) studies that provided sufficient data on hypertension and proteinuria. We excluded articles not written in English, review articles, case reports, commentaries, systematic reviews, meta-analyses, and conference abstracts.

### Outcome definition

The primary outcomes included the incidence of all-grade and grade ≥3 hypertension and proteinuria, as defined by the National Cancer Institute’s Common Terminology Criteria for Adverse Events (CTCAE). The secondary outcome included the incidence of major adverse cardiac events (MACE), including cerebrovascular accident, cardiogenic shock, heart failure, myocardial infarction, pulmonary embolism, and atrial fibrillation.

### Data extraction and quality assessment

After eliminating duplicates, Y.S. and K.T. independently reviewed the abstracts. Any differences in the results between the two investigators were resolved by discussion. If the abstract was deemed potentially relevant, the full text of the publication was reviewed by the same investigators. We extracted data, including study details (such as first author, year of publication, country, number of patients, and patient characteristics), starting dose of lenvatinib, and data on the incidence of hypertension, proteinuria, and MACE. To assess the quality of the randomized controlled trials (RCTs), we used the revised Cochrane Risk of Bias 2 (RoB 2) tool. The RoB 2 assesses the risk of bias from five domains in randomized trials: the randomization process, deviations from intended interventions, missing outcome data, outcome measurement, and selection of the reported result. For each of these domains, we reached a risk of bias judgement (low risk, some concerns, or high risk) and then made a final judgment about the entire study based on the ratings for each domain. To evaluate the quality of non-RCTs, we employed the Newcastle–Ottawa Scale (NOS),^23^ which consists of a 9-point rating scale for selection, comparability, and exposure. We rated articles with a score > 6 as “high” quality, ≤ 6 but > 4 as “moderate” quality, and ≤ 4 as “low” quality; low quality articles were not included as previously described.^24^

### Statistical analysis

For all the selected studies, the number of patients who developed all-grade and grade ≥3 hypertension or proteinuria, as well as the number of patients who received lenvatinib, were pooled. For the calculation of incidence, the number of patients who developed adverse events—such as hypertension and proteinuria—and the total number of patients treated with lenvatinib were extracted from the selected studies. The proportion of event incidence rates and their 95% exact confidence intervals (CIs) were then calculated for each study. Odds ratios (ORs) and their 95% CIs were also calculated for studies that included a control group. All the statistical analyses were performed using R version 4.0.3 (The R Foundation for Statistical Computing, Vienna, Austria) and EZR (Saitama Medical Center, Jichi Medical University, Saitama, Japan), a graphical user interface for R.^25^ Heterogeneity between studies was assessed using *I*^2^ statistics (low: 25%-50%, moderate: 50%-75%, and high: >75%). If *I*^2^ was ≥50%, a random effects model was selected; otherwise, a fixed effects model was chosen. Publication bias was assessed using funnel plots and Egger’s test. Statistically significant differences, except for the Egger’s test, were set at *P*-values <.05. For the Egger’s test, *P *< .1 was considered statistically significant.

### Ethics approval

Ethical approval was not required for this study because it involved the retrieval and synthesis of data from previously published studies.

## Results

### Search results

Figure S1 shows the flow diagram used to extract the studies for analysis in this study. A total of 5154 studies were extracted from databases based on search terms. Of these, 1800 articles were excluded because they were duplicates. Of the remaining 3354 studies, 3192 were excluded because they were review articles, case reports, and systematic reviews. Of the remaining 162 studies, 16 could not be retrieved in full text. The remaining 146 studies then underwent full-text review. Finally, 64 eligible studies were included in the analysis.

### Characteristics of the included studies

The characteristics of each study included in the analysis are shown in Table S3. A total of 64 eligible studies were identified for analysis, which contained 8115 patients treated with lenvatinib and 2328 control patients. Regarding the study design, 22 studies were prospective (including four RCTs), and the remaining 42 were retrospective. Underlying malignancies included HCC (41 studies), thyroid cancer (15 studies), biliary tract (1 study), solid tumor (1 study), renal cell carcinoma (1 study), lung cancer (1 study), thymic carcinoma (1 study), neuroendocrine neoplasm (1 study), endometrial cancer (1 study), and colorectal cancer (1 study). The starting treatment dose was classified as high-dose (≥20 mg/day) for 22 studies and as low-dose (≤12 mg/day) for 42 studies.

### Risk of bias assessment

Observational studies underwent evaluation using the NOS scale. Two of the included studies were of “moderate” quality and the remainder were “high” (Table S4). The Rob 2 tool revealed no high risk of bias in the RCTs (Figure S2).

### Hypertension

**Figure 1.**
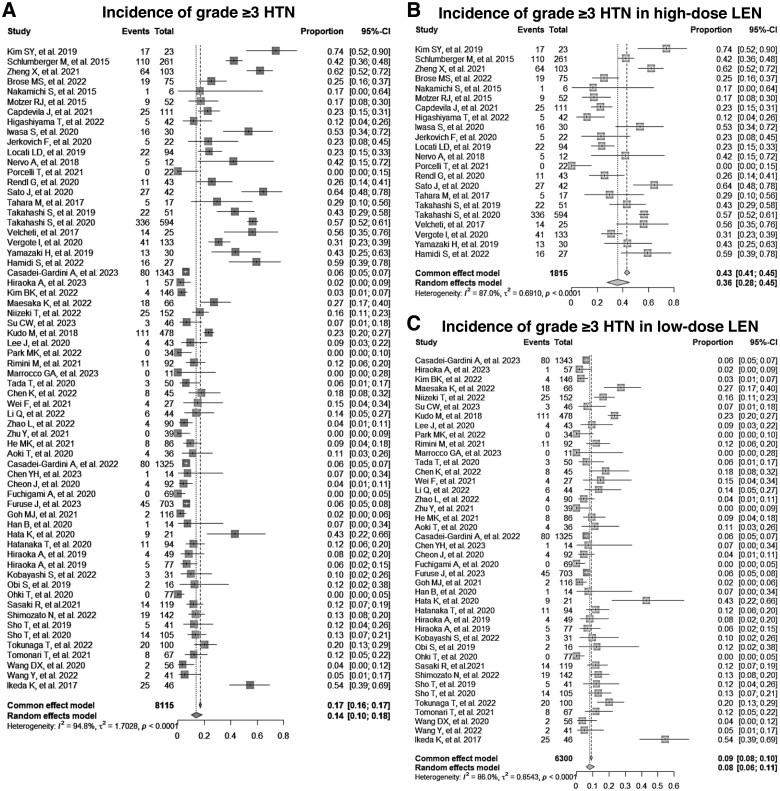
Incidence of grade ≥3 hypertension in patients receiving lenvatinib. (A) Pooled prevalence of grade ≥3 hypertension in patients receiving any dose of lenvatinib. (B) Pooled prevalence of grade ≥3 hypertension in patients receiving high-dose (≥20 mg/day) lenvatinib. (C) Pooled prevalence of grade ≥3 hypertension in patients receiving low-dose (≤12 mg/day) lenvatinib. CI = confidence interval, LEN = lenvatinib, HTN = hypertension.

**Figure 2.**
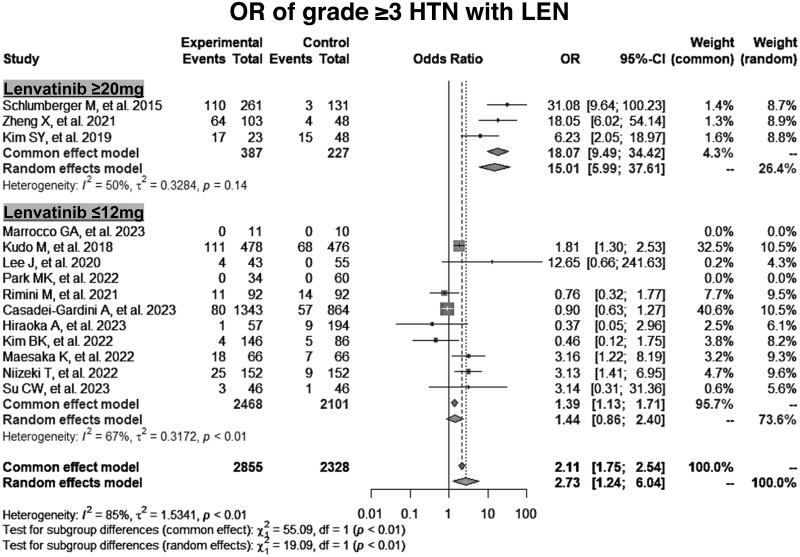
Impact of high- and low-dose lenvatinib on the incidence of grade ≥3 hypertension. CI = confidence interval, LEN = lenvatinib, OR = odds ratio, HTN = hypertension.

The pooled incidence of all-grade hypertension among patients treated with lenvatinib was 50% (95% CI, 43%-58%), and that of grade ≥3 hypertension was 14% (95% CI, 10%-18%) (Figure 1A and Figure S3A). A significant degree of heterogeneity was observed among the included studies for both all-grade and grade ≥3 hypertension (both *I*^2^ = 95%). Subgroup analyses were performed to assess potential sources of this heterogeneity (Table S5). The prevalence of all-grade and grade ≥3 hypertension in prospective studies was significantly higher than that reported in retrospective studies (both *P *< .001). Additionally, subgroup analysis by cancer type showed significant differences in the prevalence of all-grade and grade ≥3 hypertension among patients with thyroid cancer, HCC, and other cancers (both *P *< .001). The summary OR for hypertension associated with lenvatinib, compared to the control group, was calculated among the 5183 patients from 14 studies. As controls, 6 studies used atezolizumab plus bevacizumab, 6 studies used sorafenib, and the rest used placebo. The ORs for all-grade and grade ≥3 hypertension were 2.40 (95% CI, 1.41-4.09) and 2.73 (95% CI, 1.24-6.04), respectively, compared to those of the controls (Figure 2 and Figure S4), indicating a significantly increased risk of hypertension associated with lenvatinib.

**Figure 3.**
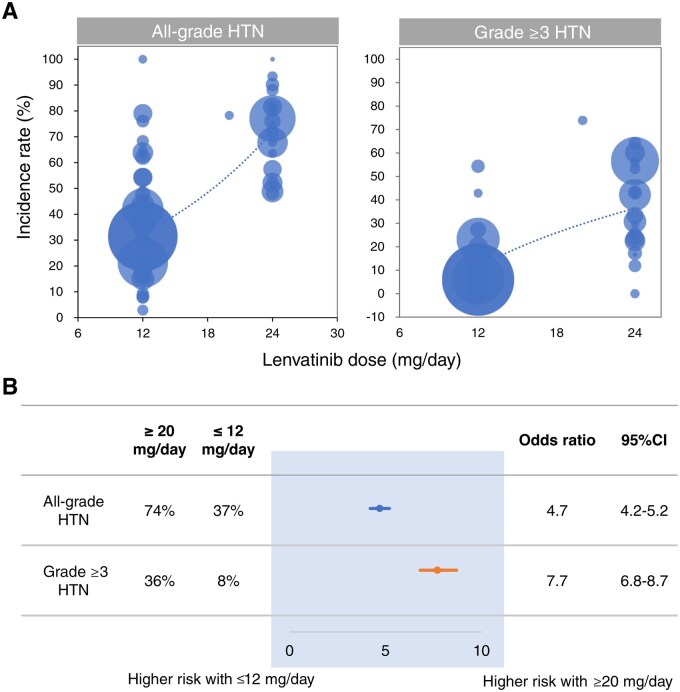
Association between lenvatinib dose and development of hypertension. (A) Bubble plots highlight possible relationships between lenvatinib dose and the incidence of hypertension. Bubble size represents the sample size of participants. (B) Odds ratio (OR) for the development of hypertension compared to low-dose lenvatinib. HTN = hypertension.

Further stratified analyses based on lenvatinib dosage were conducted to investigate the relationship between the increased risk of hypertension and lenvatinib dose. The incidence of hypertension was analyzed separately for high-dose (≥20 mg/day) and low-dose (≤12 mg/day) lenvatinib. For the high dose (≥20 mg/day), which included 1815 patients across 22 studies, the pooled prevalence of all-grade hypertension was 74% (95% CI, 67%-79%), and that of grade ≥3 hypertension was 36% (95% CI, 28%-45%) (Figure 1B, Table S5, Figure S3B). In contrast, for the low dose (≤12 mg/day) with 6300 patients across 42 studies, the prevalence of all-grade hypertension was 37% (95% CI, 30%-45%), and that of grade ≥3 hypertension was 8% (95% CI, 6%-11%) (Figure 1C, Table S5, Figure S3C). The OR for all-grade hypertension with high-dose lenvatinib compared to that of controls was 13.2 (95% CI, 5.9-29.2), whereas the OR for low-dose was 1.35 (95% CI, 0.93-1.96), as shown in Figure S4. Similarly, the OR for grade ≥3 hypertension with high-dose lenvatinib was 15.0 (95% CI, 6.0-37.6), whereas the OR for low-dose was 1.44 (95% CI, 0.86-2.40), as shown in Figure 2. Overall, the ORs comparing high-dose lenvatinib to low-dose lenvatinib were 4.7 (95% CI, 4.2-5.2) for all-grade hypertension and 7.7 (95% CI, 6.8-8.7) for grade ≥3 hypertension (Figure 3), suggesting that the risk of hypertension may be dose-dependent.

### Proteinuria

**Figure 4.**
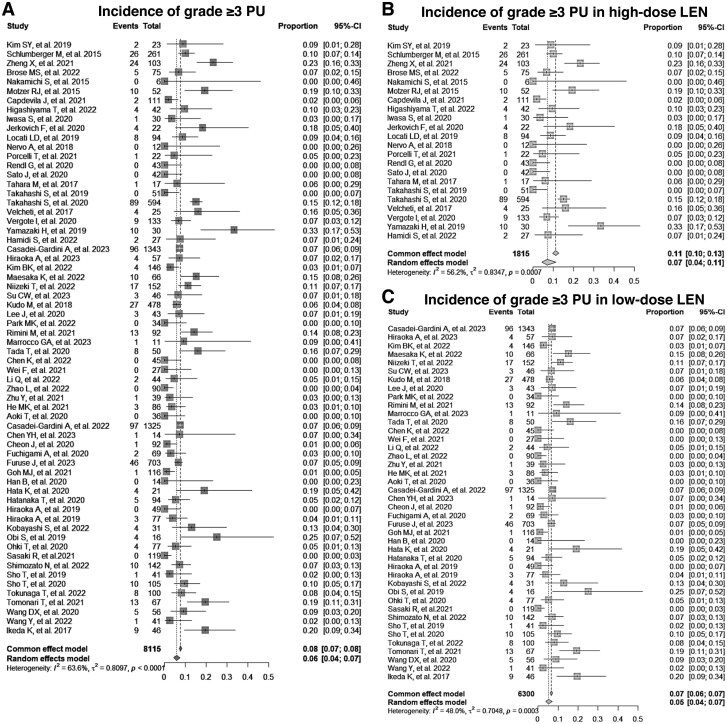
Incidence of grade ≥3 proteinuria in patients receiving lenvatinib. (A) Pooled prevalence of grade ≥3 proteinuria in patients receiving any dose of lenvatinib. (B) Pooled prevalence of grade ≥3 proteinuria in patients receiving high-dose (≥20 mg/day) lenvatinib. (C) Pooled prevalence of grade ≥3 proteinuria in patients receiving low-dose (≤12 mg/day) lenvatinib. CI = confidence interval, LEN = lenvatinib, PU = proteinuria.

**Figure 5.**
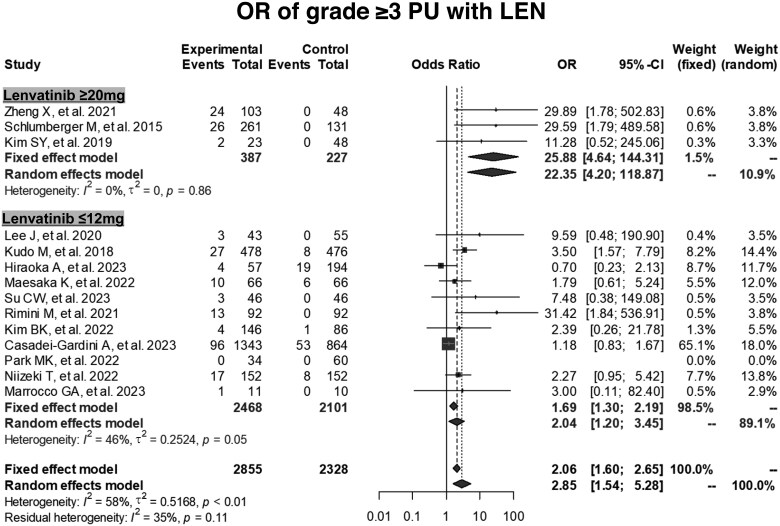
Impact of high- and low-dose lenvatinib on the incidence of grade ≥3 proteinuria. CI = confidence interval, LEN = lenvatinib, OR = odds ratio, PU = proteinuria.

The pooled incidence of all-grade proteinuria among patients treated with lenvatinib was 32% (95% CI, 26%-38%) and 6% (95% CI, 4%-7%) for grade ≥3 proteinuria (Figure 4A and Figure S5A). A high degree of heterogeneity was observed for all-grade proteinuria (*I*^2^ = 90%), whereas moderate heterogeneity was noted for grade ≥3 proteinuria (*I*^2^ = 64%). Subgroup analyses were conducted to assess the potential sources of this heterogeneity (Table S6). The prevalence of all-grade and grade ≥3 proteinuria in prospective studies was significantly higher than that in retrospective studies (*P *= .004 and .04, respectively). However, subgroup analysis by cancer type demonstrated no significant differences in the prevalence of all-grade and grade ≥3 proteinuria among patients with thyroid, hepatocellular, and other cancers (*P *= .17 and .35, respectively). The summary OR for proteinuria associated with lenvatinib, compared to the control group, was calculated among the 5183 patients from 14 studies. As controls, 6 studies used atezolizumab plus bevacizumab, 6 studies used sorafenib, and the rest used placebo. The ORs for all-grade and grade ≥3 proteinuria were 3.50 (95% CI, 1.87-6.57) and 2.85 (95% CI, 1.54-5.28), respectively, compared to those of controls, indicating a significantly increased risk of proteinuria associated with lenvatinib (Figure 5 and Figure S6).

Further stratified analyses based on lenvatinib dosage were conducted to investigate the relationship between the increased risk of proteinuria and lenvatinib dose. For the high dose (≥20 mg/day), the pooled prevalence of all-grade proteinuria was 43% (95% CI, 33%-54%), and that of grade ≥3 proteinuria was 7% (95% CI, 4%-11%) (Figure 4B, Table S6, Figure S5B). For the low dose (≤12 mg/day), the prevalence of all-grade proteinuria was 27% (95% CI, 21%-33%), and that of grade ≥3 proteinuria was 5% (95% CI, 4%-7%) (Figure 4C, Table S6, Figure S5C). The OR for all-grade proteinuria with high-dose lenvatinib compared to the control was 38.1 (95% CI, 15.2-95.9), whereas the OR for low-dose was 1.73 (95% CI, 1.06-2.84), as shown in Figure S6. Similarly, the OR for grade ≥3 proteinuria with high-dose lenvatinib compared to that of the control was 25.9 (95% CI, 4.6-144.3), whereas the OR for low-dose was 1.69 (95% CI, 1.30-2.19), as shown in Figure 5. Overall, the ORs comparing high-dose lenvatinib to low-dose lenvatinib were 1.8 (95% CI, 1.6-2.0) and 1.7 (95% CI, 1.5-2.1) for all-grade and grade ≥3 proteinuria, respectively (Figure S7).

### Major adverse cardiac events

Major adverse cardiac events was reported in 10 studies, with a pooled incidence of 2% (95% CI, 1%-5%) (Figure S8A). For the high dose, MACE was reported in 8 of 22 studies, resulting in a pooled incidence of 4% (95% CI, 2%-7%) (Figure S8B). The low dose exhibited MACE in 2 of 42 studies, yielding a pooled incidence of 1% (95% CI, 0%-2%) (Figure S8C). Although a trend toward a higher incidence of MACE at higher doses than lower doses was observed, the difference was not statistically significant (*P *= .098).

### Nephrotoxicity

Treatment-related nephrotoxicity, including elevated serum creatinine levels, renal impairment, and renal failure, was reported in 7 studies, with a pooled incidence of 4% (95% CI, 1%-14%) (Figure S9A). In high-dose lenvatinib treatments, renal impairment and renal failure were each reported in 3 of 22 studies each, resulting in a pooled incidence of 1% (95% CI, 0%-5%) and 1% (95% CI, 0%-1%), respectively (Figure S9B and S9C). Additionally, elevated serum creatinine levels were reported in 1 of 22 studies, with an incidence rate of 20%. For low doses, elevated serum creatinine levels were reported in 2 of 42 studies, with a pooled incidence of 17% (95% CI, 12%-24%) (Figure S9D).

### Publication bias

Publication bias was found in funnel plots depicting the association between lenvatinib and all-grade hypertension, grade ≥3 hypertension, or grade ≥3 proteinuria, and the association between high-dose lenvatinib and grade ≥3 hypertension or grade ≥3 proteinuria (Figure S10).

## Discussion

We performed a comprehensive meta-analysis to investigate the risks and incidence of hypertension and proteinuria associated with lenvatinib among patients with different cancers. The main findings are as follows: (1) a significant proportion of patients treated with lenvatinib experienced hypertension and proteinuria, with an incidence of 50%, 14%, 32%, and 6% for all-grade hypertension, grade ≥3 hypertension, all-grade proteinuria, and grade ≥3 proteinuria, respectively; (2) compared with controls, lenvatinib significantly increased the risk of hypertension and proteinuria; (3) compared to low-dose lenvatinib, high-dose lenvatinib treatment significantly increased the risks of hypertension and proteinuria.

Lenvatinib has been clinically validated as a targeted agent against various cancers and may result in a range of adverse effects. Given that hypertension and proteinuria are major risk factors for cardiovascular and renal events, it is particularly essential to recognize, monitor, and manage these risks promptly and appropriately. Our study is the largest meta-analysis investigating the risks and incidence of hypertension and proteinuria associated with lenvatinib, and is the first meta-analysis to examine the differences in the development of hypertension and proteinuria based on lenvatinib dosage.

The dosing of lenvatinib varies depending on the type of cancer: the starting dose of lenvatinib is 24 mg/day for thyroid cancer and thymic carcinoma, 20 mg/day for endometrial cancer, and 12 mg/day for HCC.^3–6^ The present study revealed that the incidence of hypertension and proteinuria was markedly higher in patients receiving high-dose lenvatinib (≥20 mg/day), with a 7.7-fold increased risk of grade ≥3 hypertension and a 1.7-fold increased risk of grade ≥3 proteinuria compared to those receiving low-dose lenvatinib. These results suggest that close monitoring for hypertension and proteinuria is necessary when starting lenvatinib at a dose of ≥20 mg/day. We previously reported that in thyroid cancer patients started on 24 mg/day of lenvatinib, blood pressure (BP) was already significantly elevated the day after treatment initiation, with a median time to onset of grade ≥3 hypertension of 2 days.^8^ In addition, even if baseline BP was within the normal range (systolic BP [SBP] <140 mmHg and diastolic BP [DBP] <90 mmHg), patients with normal BP (SBP 120-129 mmHg and/or DBP 80-84 mmHg) and high-normal BP (SBP 130-139 mmHg and/or DBP 85-89 mmHg) had a 5.1- and 7.5-fold higher risk of developing grade ≥3 hypertension, respectively, compared to those with optimal baseline BP (SBP <120 mmHg and DBP <80 mmHg).^8^ These results underscore the need for intensive monitoring and adjustment of antihypertensive therapy for patients receiving high-dose lenvatinib. On the other hand, while the incidence of hypertension and proteinuria was lower at initial low doses compared to high doses, the incidence of grade ≥3 hypertension (8%) and grade ≥3 proteinuria (7%) was still significant. These findings suggest that even at low doses, clinicians should remain vigilant considering the development of high-grade hypertension and proteinuria.

Lenvatinib is reportedly associated with a higher risk of cardiovascular toxicity than other VEGFR-TKIs, such as sorafenib and sunitinib.^26^ In this study, the pooled incidence of MACE was 2%, with 4% and 1% at high and low doses, respectively. The lack of a statistically significant difference between the high and low doses with respect to the occurrence of MACE may be attributed to a lack of statistical power or a short observation period.

Our meta-analysis showed that the incidence of any-grade renal impairment or renal failure was quite low, at 1%. Additionally, in our previous retrospective study of 76 patients treated with lenvatinib, we observed a significant decrease in the estimated glomerular filtration rate (eGFR) from 2 years after treatment initiation; however, eGFR improved after the end of treatment.^27^ Based on these findings, lenvatinib treatment frequently causes proteinuria but rarely leads to irreversible renal dysfunction. The evidence regarding the relationship between lenvatinib-associated proteinuria and renal dysfunction is limited and conflicting. In our previous retrospective study, the incidence of severe renal dysfunction, defined as eGFR <30 mL/min/1.73m^2^, was similar between patients with and without grade ≥3 proteinuria. Additionally, no patients in either group permanently discontinued treatment due to renal dysfunction.^27^ Fukuda et al. also reported that no significant deterioration in eGFR was observed in patients with grade 3 proteinuria compared with patients with grades 0-2 proteinuria.^28^ Conversely, Masaki et al. reported that patients with decreased eGFR after lenvatinib treatment experienced a higher incidence of grade ≥3 proteinuria compared to those without a decrease in eGFR.^29^ In this way, the relationship between lenvatinib-associated proteinuria and renal dysfunction remains unclear, and long-term studies with larger sample sizes are necessary.

There are several limitations to this study. First, although we made a considerable effort to use multiple databases to include relevant studies in our analysis, it is possible that some studies were not excluded. Nevertheless, we conducted a thorough literature search, selected studies, and extracted data to minimize risk. Second, retrospective studies may not have evaluated hypertension and proteinuria according to a defined protocol; therefore, the assessment of these adverse events may be subject to detection bias. Furthermore, due to the high heterogeneity in the prevalence of hypertension and proteinuria, the findings of this study should be interpreted with caution. Finally, certain publication bias was observed, which may limit the persuasiveness of the results. Nevertheless, we believe that the findings of this study are clinically important as they may contribute to improved management of hypertension and proteinuria in patients receiving lenvatinib.

## Conclusion

Lenvatinib treatment increases the risk of hypertension and proteinuria, particularly at high doses. Close monitoring and effective management of these adverse effects may play a vital role in the broader and safer use of lenvatinib in clinical settings. Furthermore, future studies are strongly encouraged to elucidate the mechanisms of lenvatinib-induced hypertension and proteinuria to guide therapy for these adverse effects.

## Supplementary Material

oyaf203_Supplementary_Data

## Data Availability

The data underlying this article will be shared on reasonable request to the corresponding author.
