# Supplementary material for: Incidence and risk of hypertension and proteinuria in cancer patients treated with lenvatinib: a systematic review and meta-analysis
Source: Oncologist. 2025 Jul 9;30(9):oyaf203. doi: 10.1093/oncolo/oyaf203 (PMC12449046; doi:10.1093/oncolo/oyaf203)
Supplement: oyaf203_Supplementary_Data [file oyaf203_supplementary_data.zip › Supple Figures_final.docx]

***Supplemental Figures and Figure Legends***

***
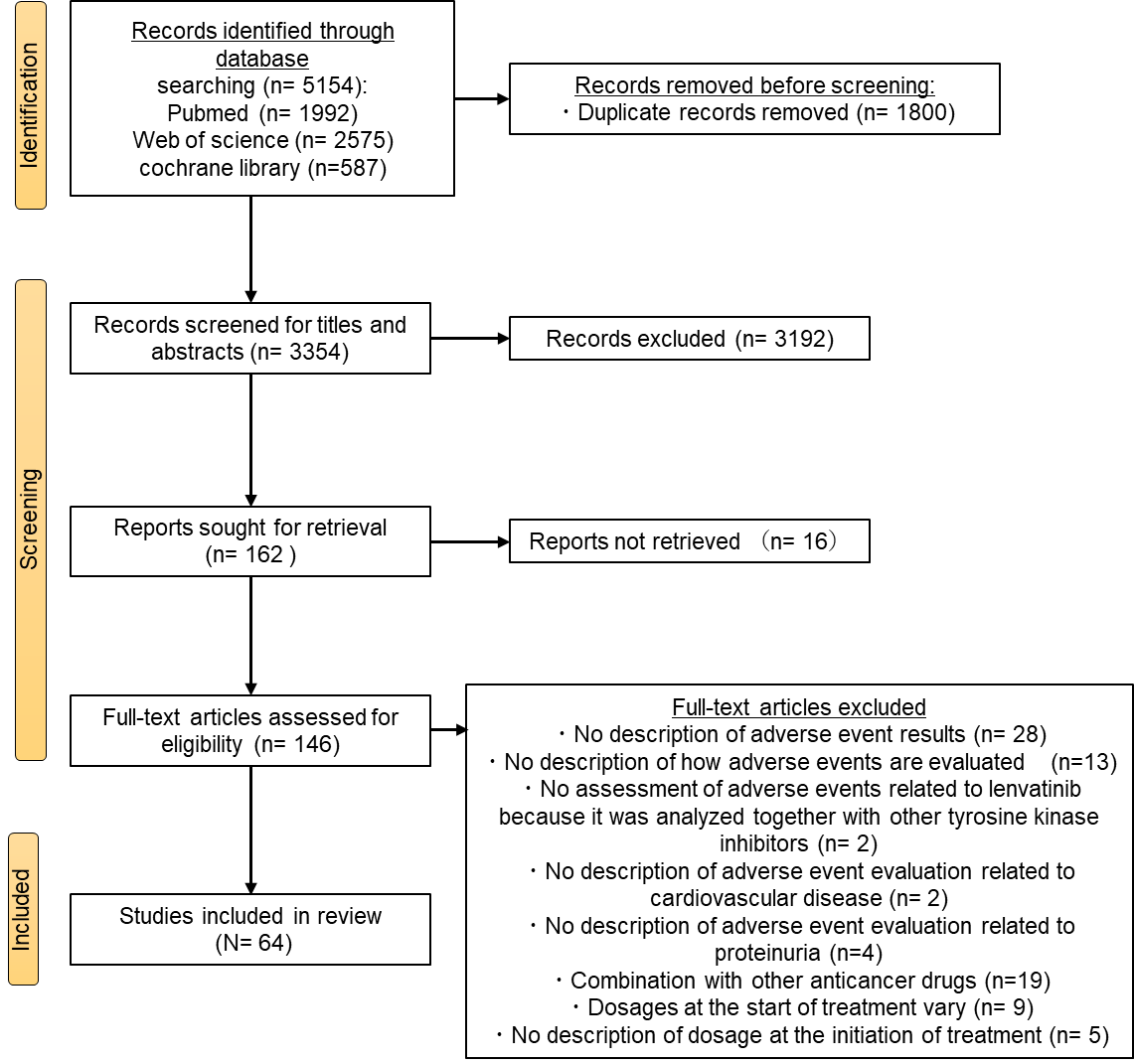
***

**Supplemental Figure 1. Preferred Reporting Items for Systematic Reviews and Meta-Analyses (PRISMA) flowchart of the study selection process.**


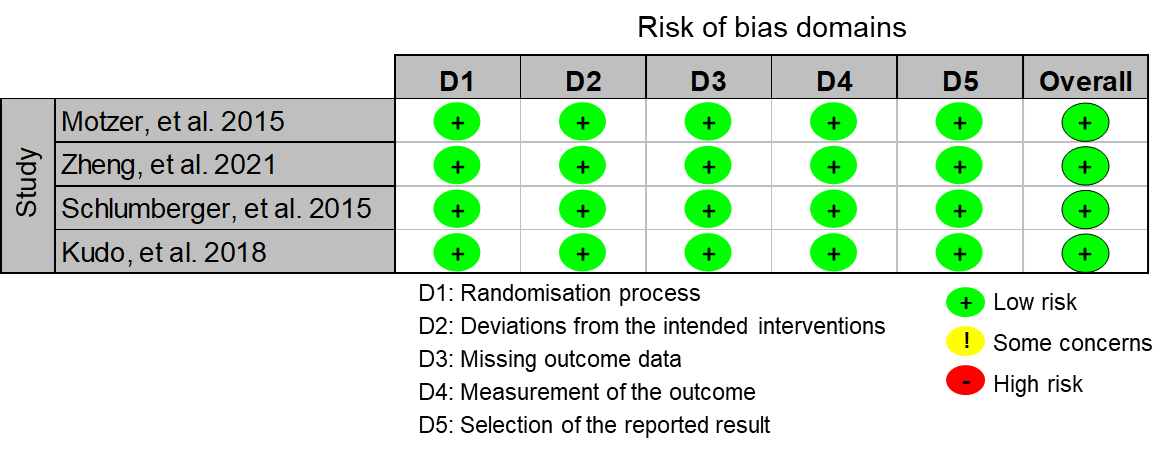


**Supplemental Figure 2. Risk of bias assessment results using the Cochrane risk of bias (Rob 2) tool for randomized clinical trial studies.**

**
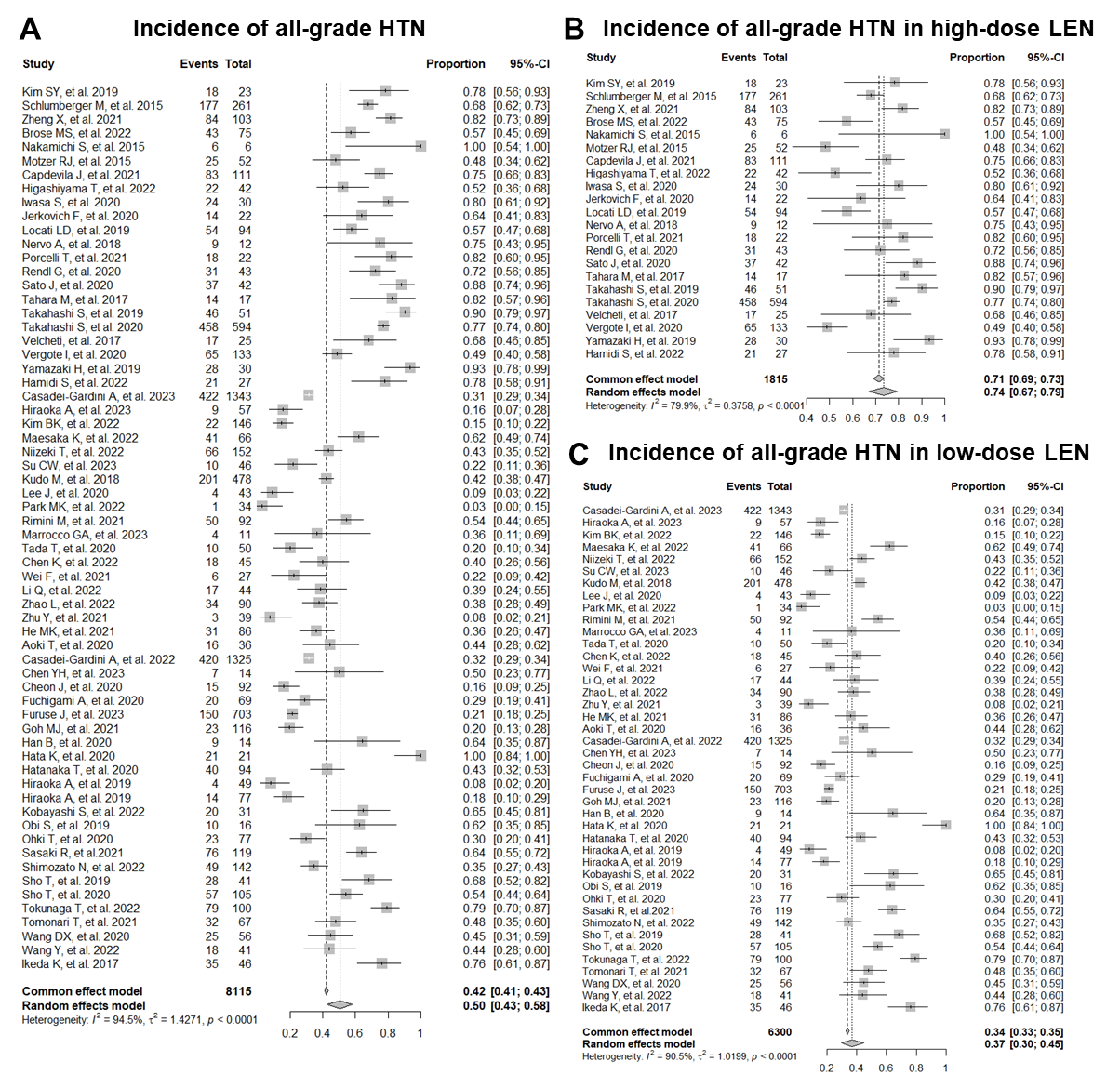
**

**Supplemental Figure 3. Incidence of all-grade hypertension in patients receiving lenvatinib.**

A, Pooled prevalence of all-grade hypertension in patients receiving any dose of lenvatinib. B, Pooled prevalence of all-grade hypertension in patients receiving high-dose (≥20 mg/day) lenvatinib. C, Pooled prevalence of all-grade hypertension in patients receiving low-dose (≤12 mg/day) lenvatinib. CI = confidence interval, LEN = lenvatinib, HTN = hypertension.

**
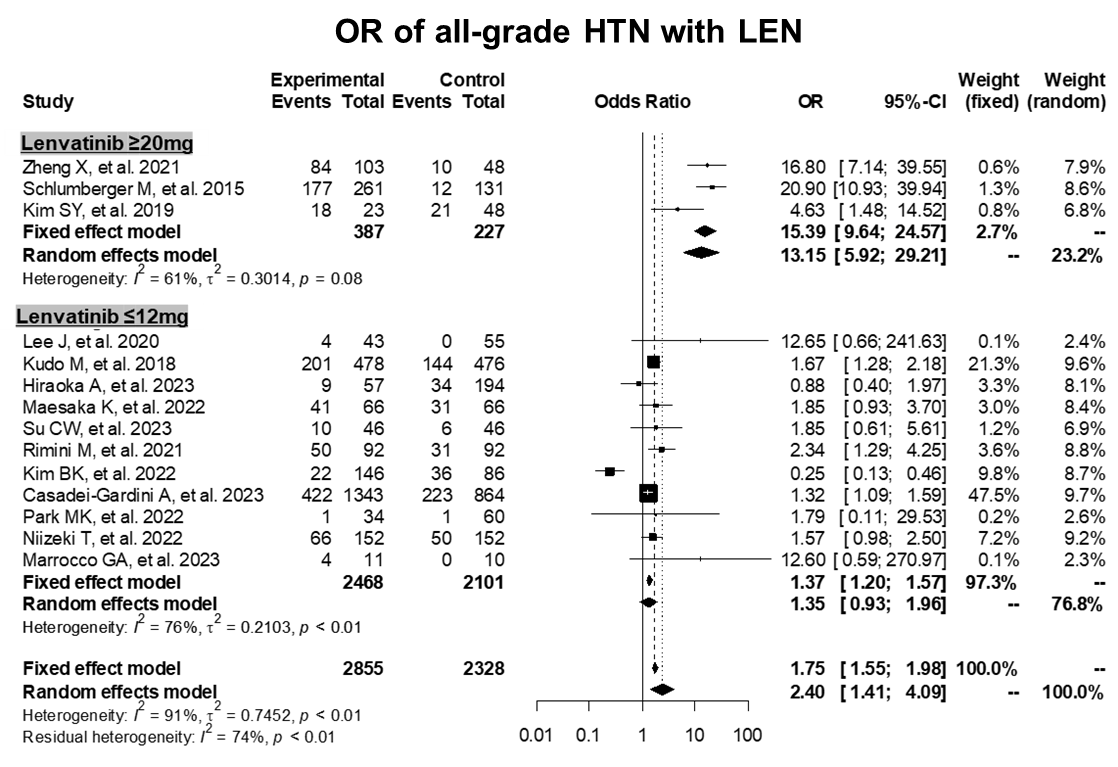
**

**Supplemental Figure 4. Impact of high- and low-dose lenvatinib on the incidence of all-grade hypertension.**

CI = confidence interval, LEN = lenvatinib, OR = odds ratio, HTN = hypertension.

**
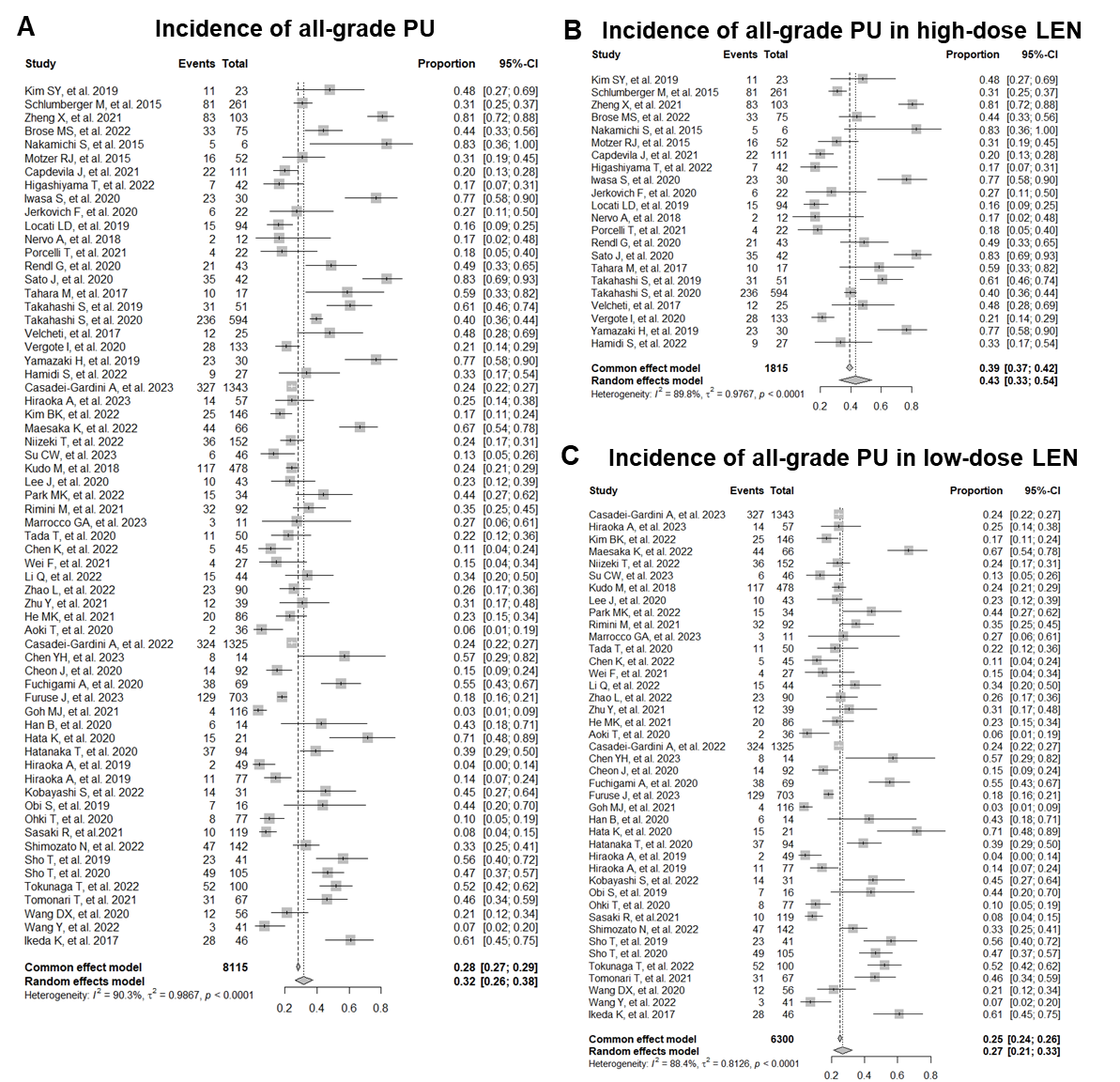
**

**Supplemental Figure 5. Incidence of all-grade proteinuria in patients receiving lenvatinib.**

A, Pooled prevalence of all-grade proteinuria in patients receiving any dose of lenvatinib. B, Pooled prevalence of all-grade proteinuria in patients receiving high-dose (≥20 mg/day) lenvatinib. C, Pooled prevalence of all-grade proteinuria in patients receiving low-dose (≤12 mg/day) lenvatinib. CI = confidence interval, LEN = lenvatinib, PU = proteinuria.

**
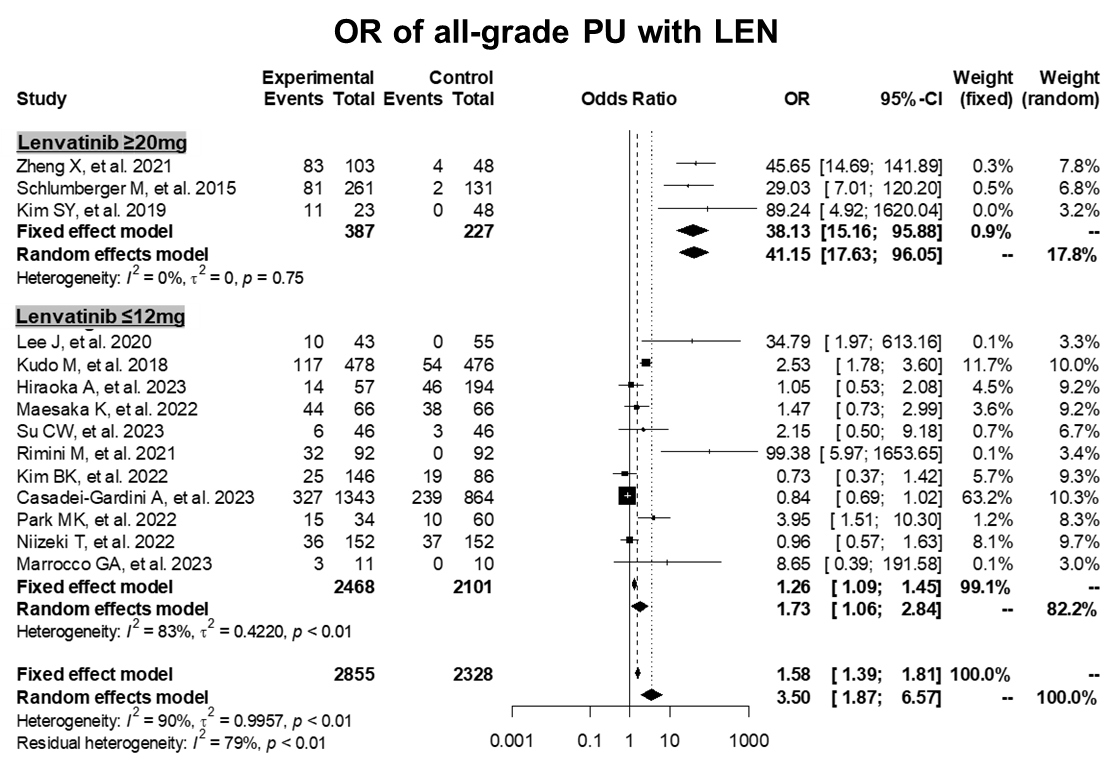
**

**Supplemental Figure 6. Impact of high- and low-dose lenvatinib on the incidence of all-grade proteinuria.**

CI = confidence interval, LEN = lenvatinib, OR = odds ratio, PU = proteinuria.


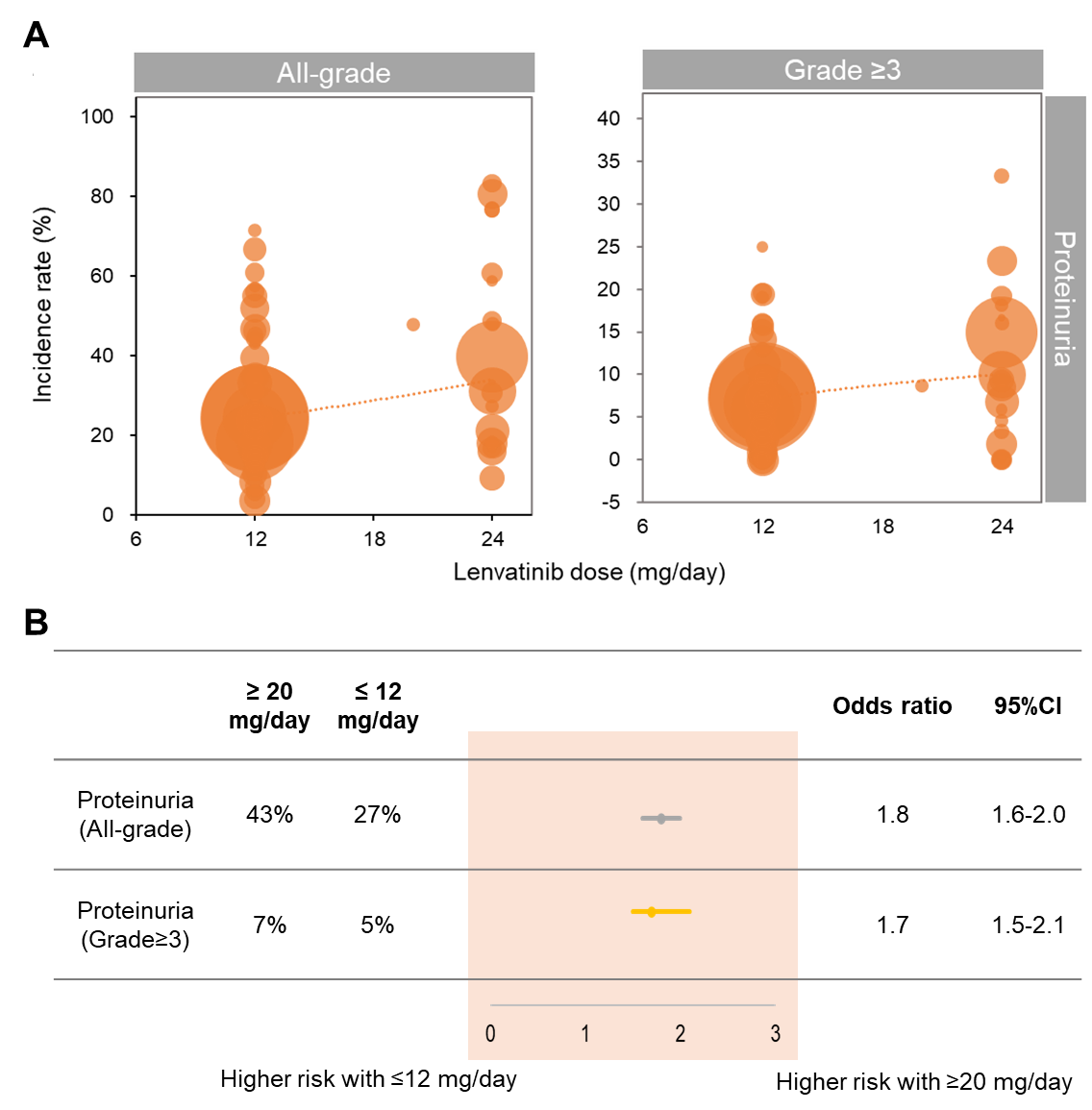


**Supplemental Figure 7. Association between lenvatinib dose and development of proteinuria.**

A, Bubble plots highlight possible relationships between lenvatinib dose and the incidence of proteinuria. Bubble size represents the sample size of participants. B, Odds ratio (OR) for the development of proteinuria compared to low-dose lenvatinib.


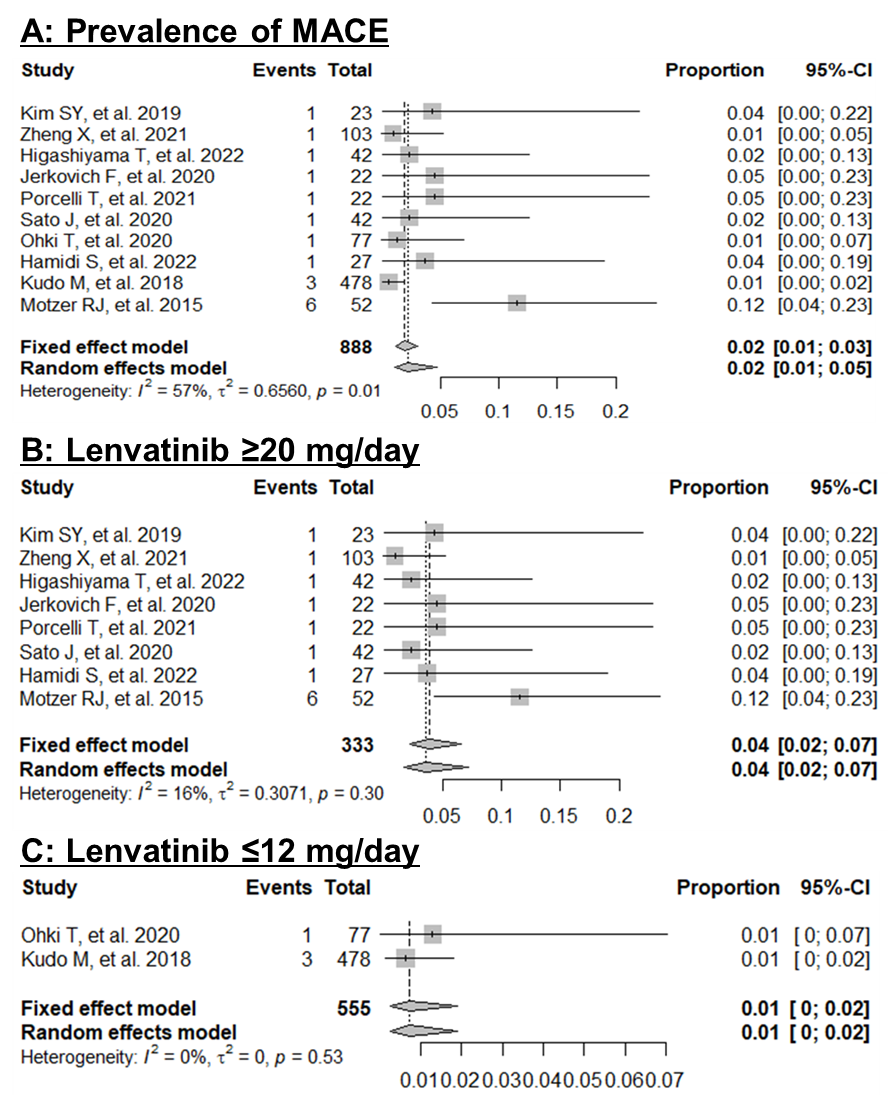


**Supplemental Figure 8. Prevalence of MACE in patients receiving lenvatinib.**

A: All, B: Lenvatinib dose ≥20 mg/day, C: Lenvatinib dose ≤12 mg/day. CI = confidence interval

**
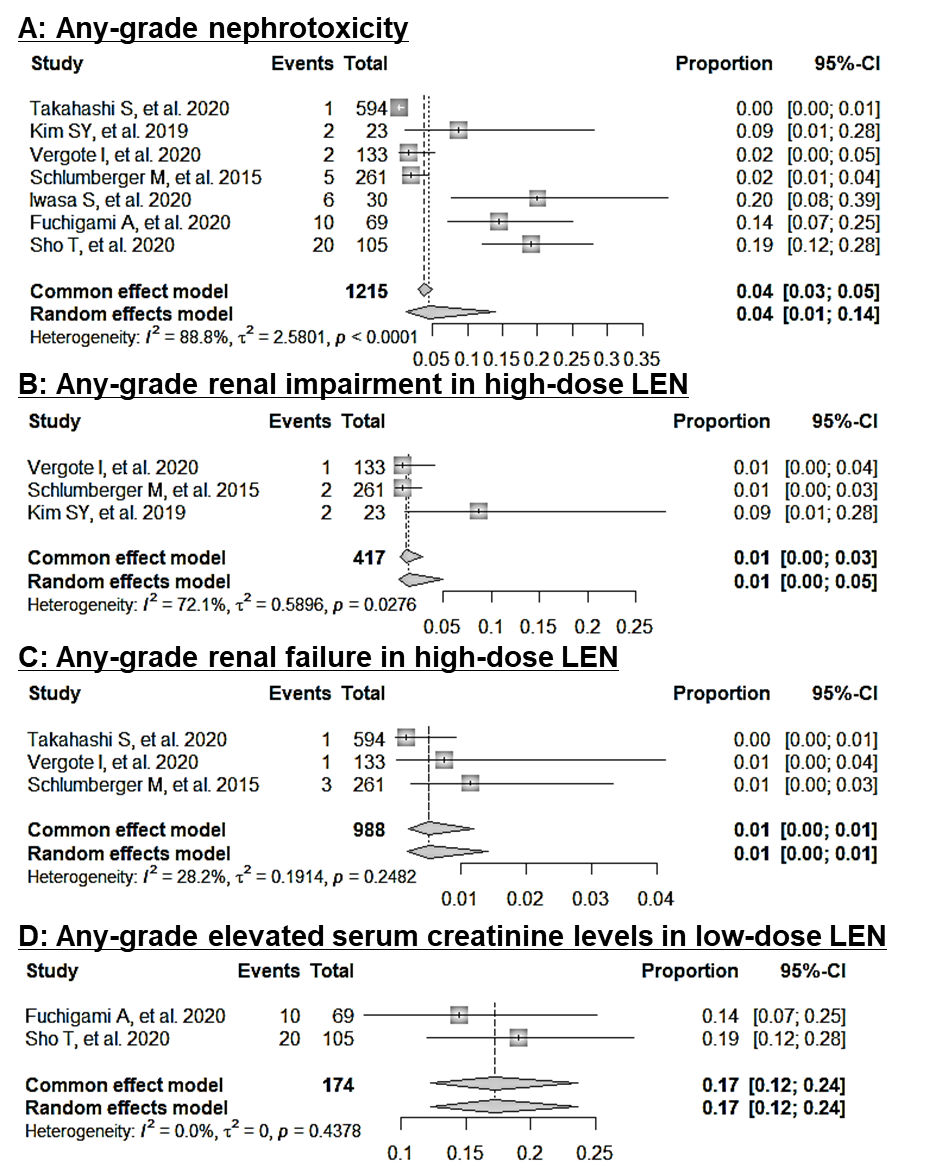
**

**Supplemental Figure 9. Prevalence of nephrotoxicity in patients receiving lenvatinib.**

A: Any-grade nephrotoxicity. B: Any-grade elevated serum creatinine levels in high-dose lenvatinib (LEN). C: Any-grade renal impairment in high-dose LEN. D: Any-grade elevated serum creatinine levels in low-dose LEN.

**
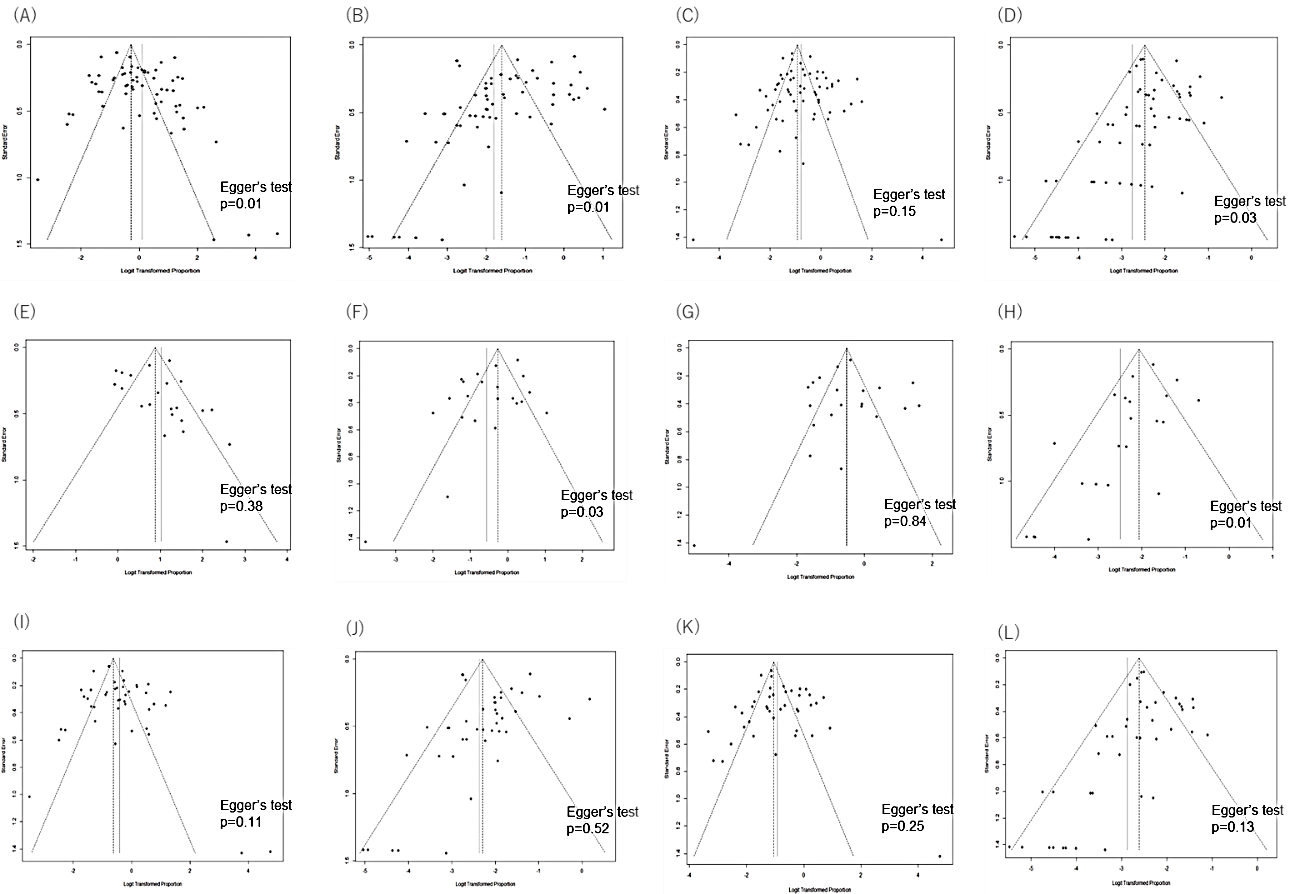
**

**Supplemental Figure 10. Funnel plots for assessing publication bias.**

Funnel plots depicting the association of lenvatinib with all-grade hypertension (A), grade ≥3 hypertension (B), all-grade proteinuria (C), and grade ≥3 proteinuria (D). Funnel plots for the association of lenvatinib dose of ≥20 mg/day with all-grade hypertension (E), grade ≥3 hypertension (F), all-grade proteinuria (G), and grade ≥3 proteinuria (H). Funnel plots for the association between lenvatinib dose of ≤20 mg/day and all-grade hypertension (I), grade ≥3 hypertension (J), all-grade proteinuria (K), and grade ≥3 proteinuria (L).
