# Supplementary material for: Incidence and risk of hypertension and proteinuria in cancer patients treated with lenvatinib: a systematic review and meta-analysis
Source: Oncologist. 2025 Jul 9;30(9):oyaf203. doi: 10.1093/oncolo/oyaf203 (PMC12449046; doi:10.1093/oncolo/oyaf203)
Supplement: oyaf203_Supplementary_Data [file oyaf203_supplementary_data.zip › Supple Tables_final.docx]

***Supplemental Tables and supporting information***

**Supplemental Table 1: PRISMA 2020 abstract checklist**

| **Section and Topic** | **Item #** | **Checklist item** | **Reported (Yes/No)** |
| --- | --- | --- | --- |
| **TITLE** | | |  |
| Title | 1 | Identify the report as a systematic review. | YES |
| **BACKGROUND** | | |  |
| Objectives | 2 | Provide an explicit statement of the main objective(s) or question(s) the review addresses. | YES |
| **METHODS** | | |  |
| Eligibility criteria | 3 | Specify the inclusion and exclusion criteria for the review. | YES |
| Information sources | 4 | Specify the information sources (e.g. databases, registers) used to identify studies and the date when each was last searched. | YES |
| Risk of bias | 5 | Specify the methods used to assess risk of bias in the included studies. | NO |
| Synthesis of results | 6 | Specify the methods used to present and synthesise results. | NO |
| **RESULTS** | | |  |
| Included studies | 7 | Give the total number of included studies and participants and summarise relevant characteristics of studies. | YES |
| Synthesis of results | 8 | Present results for main outcomes, preferably indicating the number of included studies and participants for each. If meta-analysis was done, report the summary estimate and confidence/credible interval. If comparing groups, indicate the direction of the effect (i.e. which group is favoured). | YES |
| **DISCUSSION** | | |  |
| Limitations of evidence | 9 | Provide a brief summary of the limitations of the evidence included in the review (e.g. study risk of bias, inconsistency and imprecision). | YES |
| Interpretation | 10 | Provide a general interpretation of the results and important implications. | YES |
| **OTHER** | | |  |
| Funding | 11 | Specify the primary source of funding for the review. | NO |
| Registration | 12 | Provide the register name and registration number. | NO |

**Supplemental Table 2: PRISMA 2020 checklist**

| **Section and Topic** | **Item #** | **Checklist item** | **Page** |
| --- | --- | --- | --- |
| **TITLE** | | | |
| Title | 1 | Identify the report as a systematic review. |  |
| **ABSTRACT** | | | |
| Abstract | 2 | See the PRISMA 2020 for Abstracts checklist. | 3 |
| **INTRODUCTION** | | | |
| Rationale | 3 | Describe the rationale for the review in the context of existing knowledge. | 5-6 |
| Objectives | 4 | Provide an explicit statement of the objective(s) or question(s) the review addresses. | 5-6 |
| **METHODS** | | | |
| Eligibility criteria | 5 | Specify the inclusion and exclusion criteria for the review and how studies were grouped for the syntheses. | 6-7 |
| Information sources | 6 | Specify all databases, registers, websites, organisations, reference lists and other sources searched or consulted to identify studies. Specify the date when each source was last searched or consulted. | 6 |
| Search strategy | 7 | Present the full search strategies for all databases, registers and websites, including any filters and limits used. | 6 |
| Selection process | 8 | Specify the methods used to decide whether a study met the inclusion criteria of the review, including how many reviewers screened each record and each report retrieved, whether they worked independently, and if applicable, details of automation tools used in the process. | 7 |
| Data collection process | 9 | Specify the methods used to collect data from reports, including how many reviewers collected data from each report, whether they worked independently, any processes for obtaining or confirming data from study investigators, and if applicable, details of automation tools used in the process. | 7 |
| Data items | 10a | List and define all outcomes for which data were sought. Specify whether all results that were compatible with each outcome domain in each study were sought (e.g. for all measures, time points, analyses), and if not, the methods used to decide which results to collect. | 6 |
|  | 10b | List and define all other variables for which data were sought (e.g. participant and intervention characteristics, funding sources). Describe any assumptions made about any missing or unclear information. | 6-7 |
| Study risk of bias assessment | 11 | Specify the methods used to assess risk of bias in the included studies, including details of the tool(s) used, how many reviewers assessed each study and whether they worked independently, and if applicable, details of automation tools used in the process. | 8 |
| Effect measures | 12 | Specify for each outcome the effect measure(s) (e.g. risk ratio, mean difference) used in the synthesis or presentation of results. | 7-8 |
| Synthesis methods | 13a | Describe the processes used to decide which studies were eligible for each synthesis (e.g. tabulating the study intervention characteristics and comparing against the planned groups for each synthesis (item #5)). | 8 |
|  | 13b | Describe any methods required to prepare the data for presentation or synthesis, such as handling of missing summary statistics, or data conversions. | 7-8 |
|  | 13c | Describe any methods used to tabulate or visually display results of individual studies and syntheses. | 8 |
|  | 13d | Describe any methods used to synthesize results and provide a rationale for the choice(s). If meta-analysis was performed, describe the model(s), method(s) to identify the presence and extent of statistical heterogeneity, and software package(s) used. | 8 |
|  | 13e | Describe any methods used to explore possible causes of heterogeneity among study results (e.g. subgroup analysis, meta-regression). | 8 |
|  | 13f | Describe any sensitivity analyses conducted to assess robustness of the synthesized results. | 8 |
| Reporting bias assessment | 14 | Describe any methods used to assess risk of bias due to missing results in a synthesis (arising from reporting biases). | 8 |
| Certainty assessment | 15 | Describe any methods used to assess certainty (or confidence) in the body of evidence for an outcome. | 7-8 |
| **RESULTS** | | | |
| Study selection | 16a | Describe the results of the search and selection process, from the number of records identified in the search to the number of studies included in the review, ideally using a flow diagram. | 8-9 |
|  | 16b | Cite studies that might appear to meet the inclusion criteria, but which were excluded, and explain why they were excluded. | 8-9 |
| Study characteristics | 17 | Cite each included study and present its characteristics. | 9 |
| Risk of bias in studies | 18 | Present assessments of risk of bias for each included study. | 9 |
| Results of individual studies | 19 | For all outcomes, present, for each study: (a) summary statistics for each group (where appropriate) and (b) an effect estimate and its precision (e.g. confidence/credible interval), ideally using structured tables or plots. | 9-12 |
| Results of syntheses | 20a | For each synthesis, briefly summarise the characteristics and risk of bias among contributing studies. | 9-12 |
|  | 20b | Present results of all statistical syntheses conducted. If meta-analysis was done, present for each the summary estimate and its precision (e.g. confidence/credible interval) and measures of statistical heterogeneity. If comparing groups, describe the direction of the effect. | 9-12 |
|  | 20c | Present results of all investigations of possible causes of heterogeneity among study results. | 9-10 |
|  | 20d | Present results of all sensitivity analyses conducted to assess the robustness of the synthesized results. | 9-12 |
| Reporting biases | 21 | Present assessments of risk of bias due to missing results (arising from reporting biases) for each synthesis assessed. | 12 |
| Certainty of evidence | 22 | Present assessments of certainty (or confidence) in the body of evidence for each outcome assessed. | 12 |
| **DISCUSSION** | | | |
| Discussion | 23a | Provide a general interpretation of the results in the context of other evidence. | 12-14 |
|  | 23b | Discuss any limitations of the evidence included in the review. | 12-14 |
|  | 23c | Discuss any limitations of the review processes used. | 14 |
|  | 23d | Discuss implications of the results for practice, policy, and future research. | 12-14 |
| **OTHER INFORMATION** | | | |
| Registration and protocol | 24a | Provide registration information for the review, including register name and registration number, or state that the review was not registered. | NA |
|  | 24b | Indicate where the review protocol can be accessed, or state that a protocol was not prepared. | NA |
|  | 24c | Describe and explain any amendments to information provided at registration or in the protocol. | NA |
| Support | 25 | Describe sources of financial or non-financial support for the review, and the role of the funders or sponsors in the review. | 1 |
| Competing interests | 26 | Declare any competing interests of review authors. | 15 |
| Availability of data, code and other materials | 27 | Report which of the following are publicly available and where they can be found: template data collection forms; data extracted from included studies; data used for all analyses; analytic code; any other materials used in the review. | 15 |

**Supplemental Table 3: Characteristics of the studies included in this meta-analysis**

| **Study** | **Study design** | **Type of cancer** | **Starting dose** | **No. of patients** | **No. of all-grade hypertension** | **No. of grade ≥3 hypertension** | **No. of all-grade proteinuria** | **No. of grade ≥3 proteinuria** | **Country** |
| --- | --- | --- | --- | --- | --- | --- | --- | --- | --- |
| Brose, et al. 2022^1^ | Prospective cohort | Thyroid cancer | 24 | 75 | 57 | 25 | 7 | 7 | USA |
| Nakamichi, et al. 2015^2^ | Prospective cohort | Solid tumor | 24 | 6 | 6 | 1 | 2 | 1 | Japan |
| Motzer, et al. 2015^3^ | RCT | Renal cell carcinoma | 24 | 52 | 25 | 9 | 16 | 10 | Multiple countries |
| Zheng, et al. 2021^4^ | RCT | Thyroid cancer | 24 | 103 | 84 | 62 | 83 | 24 | China |
| Schlumberger, et al. 2015^5^ | RCT | Thyroid cancer | 24 | 261 | 177 | 110 | 81 | 26 | Multiple countries |
| Tahara, et al. 2017^6^ | Prospective cohort | Thyroid cancer | 24 | 17 | 14 | 5 | 10 | 1 | Japan |
| Higashiyama, et al. 2022^7^ | Prospective cohort | Thyroid cancer | 24 | 42 | 22 | 5 | 7 | 4 | Japan |
| Nervo, et al. 2018^8^ | Retrospective cohort | Thyroid cancer | 24 | 12 | 9 | 5 | 2 | 0 | Italy |
| Locati, et al. 2019^9^ | Retrospective cohort | Thyroid cancer | 24 | 94 | 54 | 22 | 15 | 8 | Italy |
| Jerkovich, et al. 2020^10^ | Retrospective cohort | Thyroid cancer | 24 | 22 | 14 | 5 | 6 | 4 | Argentina |
| Rendl, et al. 2020^11^ | Retrospective cohort | Thyroid cancer | 24 | 43 | 31 | 11 | 21 | 0 | Austria |
| Porcelli, et al. 2021^12^ | Retrospective cohort | Thyroid cancer | 24 | 22 | 18 | 0 | 4 | 1 | Italy |
| Takahashi, et al. 2019^13^ | Prospective cohort | Thyroid cancer | 24 | 51 | 46 | 22 | 31 | 0 | Japan |
| Yamazaki, et al. 2019^14^ | Retrospective cohort | Thyroid cancer | 24 | 30 | 28 | 13 | 23 | 10 | Japan |
| Takahashi, et al. 2020^15^ | Prospective, post-marketing observational study | Thyroid cancer | 24 | 594 | 458 | 336 | 236 | 89 | Japan |
| Velcheti, et al. 2017^16^ | Prospective cohort | Lung cancer | 24 | 25 | 17 | 14 | 12 | 4 | USA |
| Sato, et al. 2020^17^ | Prospective cohort | Thymic cancer | 24 | 42 | 37 | 27 | 35 | 0 | Japan |
| Vergote, et al. 2020^18^ | Prospective cohort | Endometrial cancer | 24 | 133 | 65 | 41 | 28 | 9 | Multiple countries |
| Capdevila, et al. 2021^19^ | Prospective cohort | Neuroendocrine neoplasm | 24 | 111 | 58 | 25 | 20 | 2 | Multiple countries |
| Iwasa, et al. 2020^20^ | Prospective cohort | Colorectal | 24 | 30 | 24 | 16 | 23 | 1 | Japan |
| Hamidi, et al. 2022^21^ | Retrospective cohort | Thyroid cancer | 24 or 20 | 27 | 21 | 16 | 9 | 2 | Canada |
| Kim, et al. 2019^22^ | Retrospective cohort | Thyroid cancer | 20 | 23 | 18 | 17 | 11 | 2 | Korea |
| Maesaka, et al. 2022^23^ | Prospective cohort | HCC | 12 | 66 | 41 | 18 | 44 | 10 | Japan |
| Kim, et al. 2022^24^ | Retrospective cohort | HCC | 12 | 146 | 22 | 4 | 25 | 4 | Korea |
| Hiraoka, et al. 2023^25^ | Retrospective cohort | HCC | 12 | 57 | 9 | 1 | 14 | 4 | Japan |
| Niizeki, et al. 2022^26^ | Retrospective cohort | HCC | 12 | 152 | 66 | 25 | 36 | 17 | Japan |
| Su, et al. 2023^27^ | Retrospective cohort | HCC | 12 | 46 | 10 | 3 | 6 | 3 | Chaina |
| Casadei-Gardini, et al. 2023^28^ | Retrospective cohort | HCC | 12 | 1343 | 422 | 80 | 327 | 96 | Multiple countries |
| Tada, et al. 2020^29^ | Retrospective cohort | HCC | 12 | 50 | 10 | 3 | 11 | 8 | Japan |
| Wei, et al. 2021^30^ | Retrospective cohort | HCC | 12 | 27 | 6 | 4 | 4 | 0 | China |
| Chen, et al. 2022^31^ | Retrospective cohort | HCC | 12 | 45 | 18 | 8 | 5 | 0 | China |
| Zhu, et al. 2021^32^ | Retrospective cohort | HCC | 12 | 39 | 3 | 0 | 12 | 1 | China |
| Li, et al. 2022^33^ | Retrospective cohort | HCC | 12 | 44 | 17 | 6 | 15 | 2 | China |
| Zhao, et al. 2022^34^ | Retrospective cohort | HCC | 12 | 90 | 34 | 4 | 23 | 0 | China |
| He, et al. 2021^35^ | Retrospective cohort | HCC | 12 | 86 | 31 | 8 | 20 | 3 | China |
| Kudo, et al. 2018^36^ | RCT | HCC | 12 | 478 | 201 | 111 | 117 | 27 | Multiple countries |
| Rimini, et al. 2021^37^ | Prospective cohort | HCC | 12 | 92 | 50 | 11 | 32 | 13 | Multiple countries |
| Lee, et al. 2020^38^ | Retrospective cohort | HCC | 12 | 43 | 4 | 4 | 10 | 3 | Korea |
| Park, et al. 2022^39^ | Retrospective cohort | HCC | 12 | 34 | 1 | 0 | 15 | 0 | Korea |
| Marrocco, et al. 2023^40^ | Retrospective cohort | HCC | 12 | 11 | 4 | 0 | 3 | 1 | Italy |
| Hata, et al. 2020^41^ | Prospective cohort | HCC | 12 | 21 | 21 | 9 | 15 | 4 | Japan |
| Kobayashi, et al. 2022^42^ | Prospective cohort | HCC | 12 | 31 | 20 | 3 | 14 | 4 | Japan |
| Ikeda, et al. 2017^43^ | Prospective cohort | HCC | 12 | 46 | 35 | 25 | 28 | 9 | Japan |
| Tokunaga, et al. 2022^44^ | Retrospective cohort | HCC | 12 | 100 | 79 | 20 | 52 | 8 | Japan |
| Hiraoka, et al. 2019^45^ | Retrospective cohort | HCC | 12 | 49 | 4 | 4 | 2 | 0 | Japan |
| Hiraoka, et al. 2019^46^ | Retrospective cohort | HCC | 12 | 77 | 14 | 5 | 11 | 3 | Japan |
| Obi, et al. 2019^47^ | Retrospective cohort | HCC | 12 | 16 | 10 | 2 | 7 | 4 | Japan |
| Hatanaka, et al. 2020^48^ | Retrospective cohort | HCC | 12 | 94 | 40 | 11 | 37 | 5 | Japan |
| Ohki, et al. 2020^49^ | Retrospective cohort | HCC | 12 | 77 | 23 | 0 | 8 | 4 | Japan |
| Sho, et al. 2019^50^ | Retrospective cohort | HCC | 12 | 41 | 28 | 5 | 23 | 1 | Japan |
| Fuchigami, et al. 2020^51^ | Retrospective cohort | HCC | 12 | 69 | 20 | 0 | 38 | 2 | Japan |
| Sho, et al. 2020^52^ | Retrospective cohort | HCC | 12 | 105 | 57 | 14 | 49 | 10 | Japan |
| Wang, et al. 2020^53^ | Retrospective cohort | HCC | 12 | 56 | 25 | 2 | 12 | 5 | China |
| Cheon, et al. 2020^54^ | Retrospective cohort | HCC | 12 | 92 | 15 | 4 | 14 | 1 | Korea |
| Aoki, et al. 2020^55^ | Retrospective cohort | HCC | 12 | 36 | 16 | 4 | 2 | 0 | Japan |
| Han, et al. 2020^56^ | Retrospective cohort | HCC | 12 | 14 | 9 | 1 | 6 | 0 | China |
| Goh, et al. 2021^57^ | Retrospective cohort | HCC | 12 | 116 | 23 | 2 | 4 | 1 | Korea |
| Sasaki, et al.2021^58^ | Retrospective cohort | HCC | 12 | 119 | 76 | 14 | 10 | 0 | Japan |
| Tomonari, et al. 2021^59^ | Retrospective cohort | HCC | 12 | 67 | 32 | 8 | 31 | 13 | Japan |
| Shimozato, et al. 2022^60^ | Retrospective cohort | HCC | 12 | 142 | 51 | 19 | 47 | 10 | Japan |
| Casadei-Gardini, et al. 2022^61^ | Retrospective cohort | HCC | 12 | 1325 | 420 | 80 | 324 | 100 | Multiple countries |
| Furuse, et al. 2023^62^ | Prospective, observational post-marketing study | HCC | 12 | 703 | 150 | 45 | 129 | 46 | Japan |
| Chen, et al. 2023^63^ | Retrospective cohort | HCC | 12 | 14 | 7 | 1 | 8 | 1 | China |
| Wang, et al. 2022^64^ | Prospective cohort | Biliary tract cancer | 12 | 41 | 18 | 2 | 3 | 1 | China |

HCC, hepatocellular carcinoma; RCT, randomized controlled trial.

**Supplemental Table 4: Newcastle-Ottawa Scale (NOS) results for cohort study**

| Author, year | Selection | | | | Comparability | | Outcome | | | Total |
| --- | --- | --- | --- | --- | --- | --- | --- | --- | --- | --- |
|  | Representativeness of the exposed cohort | Selection of the non-exposed cohort | Ascertainment of exposure | Outcome of interest not present at start of the study | Control for primary confounders | Control for secondary confounders | Assessment of outcome | Duration of follow-up | Adequacy of follow-up |  |
| Ikeda, et al. 2017^43^ | 1 | 0 | 1 | 1 | 1 | 1 | 1 | 1 | 1 | 8 |
| Kim, et al. 2019^22^ | 1 | 1 | 1 | 1 | 1 | 1 | 1 | 1 | 1 | 9 |
| Brose, et al. 2022^1^ | 1 | 1 | 1 | 1 | 1 | 0 | 1 | 1 | 1 | 8 |
| Zheng, et al. 2021^4^ | 1 | 1 | 1 | 1 | 1 | 1 | 1 | 1 | 1 | 9 |
| Schlumberger, et al. 2015^5^ | 1 | 1 | 1 | 1 | 1 | 1 | 1 | 1 | 1 | 9 |
| Tahara, et al. 2017^6^ | 1 | 0 | 1 | 1 | 1 | 0 | 1 | 1 | 1 | 7 |
| Higashiyama, et al. 2022^7^ | 1 | 0 | 1 | 1 | 1 | 1 | 1 | 1 | 1 | 8 |
| Takahashi, et al. 2019^13^ | 1 | 0 | 1 | 1 | 1 | 0 | 1 | 1 | 1 | 7 |
| Takahashi, et al. 2020^15^ | 1 | 0 | 1 | 1 | 1 | 1 | 1 | 1 | 1 | 8 |
| Nervo, et al. 2018^8^ | 1 | 1 | 1 | 1 | 1 | 0 | 1 | 1 | 1 | 8 |
| Locati, et al. 2019^9^ | 1 | 0 | 1 | 0 | 1 | 0 | 1 | 1 | 1 | 6 |
| Jerkovich, et al. 2020^10^ | 1 | 0 | 1 | 1 | 1 | 0 | 1 | 1 | 1 | 7 |
| Rendl, et al. 2020^11^ | 1 | 0 | 1 | 1 | 1 | 0 | 1 | 1 | 1 | 7 |
| Porcelli, et al. 2021^12^ | 1 | 0 | 1 | 1 | 1 | 0 | 1 | 1 | 1 | 7 |
| Yamazaki, et al. 2019^14^ | 1 | 1 | 1 | 1 | 1 | 0 | 1 | 1 | 1 | 8 |
| Nakamichi, et al. 2015^2^ | 1 | 0 | 1 | 1 | 1 | 0 | 1 | 1 | 1 | 7 |
| Motzer, et al. 2015^3^ | 1 | 1 | 1 | 1 | 1 | 1 | 1 | 1 | 1 | 9 |
| Velcheti, et al. 2017^16^ | 1 | 0 | 1 | 0 | 1 | 1 | 1 | 1 | 1 | 7 |
| Sato, et al. 2020^17^ | 1 | 0 | 1 | 1 | 1 | 1 | 1 | 1 | 1 | 8 |
| Vergote, et al. 2020^18^ | 1 | 0 | 1 | 1 | 1 | 0 | 1 | 1 | 1 | 7 |
| Capdevila, et al. 2021^19^ | 1 | 0 | 1 | 1 | 1 | 0 | 1 | 1 | 1 | 7 |
| Iwasa, et al. 2020^20^ | 1 | 0 | 1 | 1 | 1 | 0 | 1 | 1 | 1 | 7 |
| Maesaka, et al. 2022^23^ | 1 | 1 | 1 | 1 | 1 | 1 | 1 | 1 | 1 | 9 |
| Tada, et al. 2020^29^ | 1 | 1 | 1 | 1 | 1 | 0 | 1 | 1 | 1 | 8 |
| Kudo, et al. 2018^36^ | 1 | 1 | 1 | 1 | 1 | 1 | 1 | 1 | 1 | 9 |
| Rimini, et al. 2021^37^ | 1 | 1 | 1 | 1 | 1 | 1 | 1 | 1 | 1 | 9 |
| Hata, et al. 2020^41^ | 1 | 0 | 1 | 1 | 1 | 0 | 1 | 1 | 1 | 7 |
| Kobayashi, et al. 2022^42^ | 1 | 0 | 1 | 1 | 1 | 1 | 1 | 1 | 1 | 8 |
| Kim, et al. 2022^24^ | 1 | 1 | 1 | 1 | 1 | 1 | 1 | 1 | 1 | 9 |
| Hiraoka, et al. 2023^25^ | 1 | 1 | 1 | 1 | 1 | 1 | 1 | 1 | 1 | 9 |
| Niizeki, et al. 2022^26^ | 1 | 1 | 1 | 1 | 1 | 1 | 1 | 1 | 1 | 9 |
| Su, et al. 2023^27^ | 1 | 1 | 1 | 1 | 1 | 1 | 1 | 1 | 1 | 9 |
| Casadei-Gardini, et al. 2023^28^ | 1 | 1 | 1 | 1 | 1 | 1 | 1 | 1 | 1 | 9 |
| Wei, et al. 2021^30^ | 1 | 1 | 1 | 1 | 1 | 1 | 1 | 1 | 1 | 9 |
| Chen, et al. 2022^31^ | 1 | 1 | 1 | 1 | 1 | 1 | 1 | 1 | 1 | 9 |
| Zhu, et al. 2021^32^ | 1 | 1 | 1 | 1 | 1 | 1 | 1 | 1 | 1 | 9 |
| Li, et al. 2022^33^ | 1 | 1 | 1 | 1 | 1 | 1 | 1 | 1 | 1 | 9 |
| Zhao, et al. 2022^34^ | 1 | 1 | 1 | 1 | 1 | 1 | 1 | 1 | 1 | 9 |
| He, et al. 2021^35^ | 1 | 1 | 1 | 1 | 1 | 1 | 1 | 1 | 1 | 9 |
| Lee, et al. 2020^38^ | 1 | 1 | 1 | 1 | 1 | 0 | 1 | 1 | 1 | 8 |
| Park, et al. 2022^39^ | 1 | 1 | 1 | 1 | 1 | 0 | 1 | 1 | 1 | 8 |
| Marrocco, et al. 2023^40^ | 1 | 1 | 1 | 1 | 1 | 1 | 1 | 1 | 1 | 9 |
| Tokunaga, et al. 2022^44^ | 1 | 0 | 1 | 1 | 1 | 1 | 1 | 1 | 1 | 8 |
| Hiraoka, et al. 2019^45^ | 1 | 0 | 1 | 0 | 1 | 0 | 1 | 1 | 1 | 6 |
| Hiraoka, et al. 2019^46^ | 1 | 1 | 1 | 0 | 1 | 0 | 1 | 1 | 1 | 7 |
| Obi, et al. 2019^47^ | 1 | 0 | 1 | 1 | 1 | 0 | 1 | 1 | 1 | 7 |
| Hatanaka, et al. 2020^48^ | 1 | 0 | 1 | 1 | 1 | 1 | 1 | 1 | 1 | 8 |
| Ohki, et al. 2020^49^ | 1 | 0 | 1 | 1 | 1 | 0 | 1 | 1 | 1 | 7 |
| Sho, et al. 2019^50^ | 1 | 0 | 1 | 1 | 1 | 1 | 1 | 1 | 1 | 8 |
| Fuchigami, et al. 2020^51^ | 1 | 0 | 1 | 1 | 1 | 1 | 1 | 1 | 1 | 8 |
| Sho, et al. 2020^52^ | 1 | 0 | 1 | 1 | 1 | 0 | 1 | 1 | 1 | 7 |
| Wang, et al. 2020^53^ | 1 | 0 | 1 | 1 | 1 | 0 | 1 | 1 | 1 | 7 |
| Cheon, et al. 2020^54^ | 1 | 1 | 1 | 1 | 1 | 1 | 1 | 1 | 1 | 9 |
| Aoki, et al. 2020^55^ | 1 | 0 | 1 | 1 | 1 | 1 | 1 | 1 | 1 | 8 |
| Han, et al. 2020^56^ | 1 | 0 | 1 | 1 | 1 | 0 | 1 | 1 | 1 | 7 |
| Goh, et al. 2021^57^ | 1 | 0 | 1 | 1 | 1 | 1 | 1 | 1 | 1 | 8 |
| Sasaki, et al.2021^58^ | 1 | 0 | 1 | 1 | 1 | 0 | 1 | 1 | 1 | 7 |
| Tomonari, et al. 2021^59^ | 1 | 0 | 1 | 1 | 1 | 0 | 1 | 1 | 1 | 7 |
| Shimozato, et al. 2022^60^ | 1 | 0 | 1 | 1 | 1 | 0 | 1 | 1 | 1 | 7 |
| Casadei-Gardini, et al. 2022^61^ | 1 | 0 | 1 | 1 | 1 | 1 | 1 | 1 | 1 | 8 |
| Furuse, et al. 2023^62^ | 1 | 0 | 1 | 1 | 1 | 1 | 1 | 1 | 1 | 8 |
| Chen, et al. 2023^63^ | 1 | 0 | 1 | 1 | 1 | 0 | 1 | 1 | 1 | 7 |
| Wang, et al. 2022^64^ | 1 | 0 | 1 | 1 | 1 | 1 | 1 | 1 | 1 | 8 |
| Hamidi, et al. 2022^21^ | 1 | 0 | 1 | 1 | 1 | 0 | 1 | 1 | 1 | 7 |

**Supplemental Table 5: Subgroup analyses for the prevalence of hypertension**

|  | **Subtype** | **No. of studies** | **Pooled prevalence (95% CI)** | ***I^2^* (%)** |
| --- | --- | --- | --- | --- |
| **All-grade hypertension** | Study type (p for subgroup differences <0.001) | | | |
|  | Prospective | 22 | 0.68 (0.59 - 0.76) | 91 |
|  | Retrospective | 42 | 0.40 (0.32 - 0.48) | 91 |
|  | Study type (*p* for subgroup differences <0.001) | | | |
|  | Thyroid cancer | 15 | 0.74 (0.69 - 0.80) | 75 |
|  | HCC | 41 | 0.36 (0.29 - 0.44) | 90 |
|  | Others | 8 | 0.68 (0.54 - 0.80) | 82 |
|  | Study type (p for subgroup differences <0.001) | | | |
|  | High dose (≥20 mg/day) | 22 | 0.74 (0.67-0.79) | 82 |
|  | Low dose (≤12 mg/day) | 42 | 0.37 (0.30-0.45) | 90 |
| **Grade ≥3 hypertension** | Study type (*p* for subgroup differences <0.001) | | | |
|  | Prospective | 22 | 0.29 (0.21 - 0.39) | 92 |
|  | Retrospective | 42 | 0.08 (0.06 - 0.12) | 84 |
|  | Study type (*p* for subgroup differences <0.001) | | | |
|  | Thyroid cancer | 15 | 0.35 (0.25 - 0.47) | 86 |
|  | HCC | 41 | 0.08 (0.06-0.11) | 86 |
|  | Others | 8 | 0.30 (0.16 - 0.48) | 85 |
|  | Study type (p for subgroup differences <0.001) | | | |
|  | High dose (≥20 mg/day) | 22 | 0.36 (0.28-0.45) | 86 |
|  | Low dose (≤12 mg/day) | 42 | 0.08 (0.06-0.11) | 86 |

CI, confidence interval; HCC, hepatocellular carcinoma

**Supplemental Table 6: Subgroup analyses for the prevalence of proteinuria**

|  | **Subtype** | **No. of studies** | **Pooled prevalence (95% CI)** | ***I^2^* (%)** |
| --- | --- | --- | --- | --- |
| **All-grade proteinuria** | Study type (*p* for subgroup differences = 0.004) | | | |
|  | Prospective | 22 | 0.44 (0.34 - 0.55) | 91 |
|  | Retrospective | 42 | 0.25 (0.21 - 0.31) | 86 |
|  | Study type (*p* for subgroup differences = 0.17) | | | |
|  | Thyroid cancer | 15 | 0.40 (0.29 - 0.52) | 89 |
|  | HCC | 41 | 0.27 (0.21 - 0.33) | 88 |
|  | Others | 8 | 0.43 (0.21 - 0.67) | 91 |
|  | Study type (*p* for subgroup differences = 0.025) | | | |
|  | High dose (≥20 mg/day) | 22 | 0.43 (0.33-0.54) | 89 |
|  | Low dose (≤12 mg/day) | 42 | 0.27 (0.21-0.33) | 88 |
| **Grade ≥3 proteinuria** | Study type (*p* for subgroup differences = 0.04) | | | |
|  | Prospective | 22 | 0.09 (0.06 - 0.12) | 65 |
|  | Retrospective | 42 | 0.06 (0.06 - 0.07) | 43 |
|  | Study type (*p* for subgroup differences = 0.35) | | | |
|  | Thyroid cancer | 15 | 0.12 (0.10 - 0.14) | 53 |
|  | HCC | 41 | 0.06 (0.06-0.07) | 49 |
|  | Others | 8 | 0.04 (0.01 - 0.10) | 58 |
|  | Study type (*p* for subgroup differences = 0.43) | | | |
|  | High dose (≥20 mg/day) | 22 | 0.07 (0.04-0.11) | 56 |
|  | Low dose (≤12 mg/day) | 42 | 0.05 (0.04-0.07) | 48 |

CI, confidence interval; HCC, hepatocellular carcinoma

**References**

1. Brose MS, Panaseykin Y, Konda B, de la Fouchardiere C, Hughes BGM, Gianoukakis AG, et al. A Randomized Study of lenvatinib 18 mg vs 24 mg in Patients with Radioiodine-Refractory Differentiated thyroid Cancer. *J Clin Endocrinol Metab.* 2022;107:776–787. doi: [10.1210/clinem/dgab731](https://doi.org/10.1210/clinem/dgab731).

2. Nakamichi S, Nokihara H, Yamamoto N, Yamada Y, Honda K, Tamura Y, et al. A phase 1 study of lenvatinib, multiple receptor tyrosine kinase inhibitor, in Japanese patients with advanced solid tumors. *Cancer Chemother Pharmacol.* 2015;76:1153–1161. doi: [10.1007/s00280-015-2899-0](https://doi.org/10.1007/s00280-015-2899-0).

3. Motzer RJ, Hutson TE, Glen H, Michaelson MD, Molina A, Eisen T, et al. Lenvatinib, everolimus, and the combination in patients with metastatic renal cell carcinoma: a randomised, phase 2, open-label, multicentre trial. *Lancet Oncol.* 2015;16:1473–1482. doi: [10.1016/S1470-2045(15)00290-9](https://doi.org/10.1016/S1470-2045(15)00290-9).

4. Zheng X, Xu Z, Ji Q, Ge M, Shi F, Qin J, et al. A randomized, Phase III study of lenvatinib in Chinese patients with radioiodine-refractory differentiated thyroid cancer. *Clin Cancer Res.* 2021;27:5502–5509. doi: [10.1158/1078-0432.CCR-21-0761](https://doi.org/10.1158/1078-0432.CCR-21-0761).

5. Schlumberger M, Tahara M, Wirth LJ, Robinson B, Brose MS, Elisei R, et al. Lenvatinib versus placebo in radioiodine-refractory thyroid cancer. *N Engl J Med.* 2015;372:621–630. doi: [10.1056/NEJMoa1406470](https://doi.org/10.1056/nejmoa1406470).

6. Tahara M, Kiyota N, Yamazaki T, Chayahara N, Nakano K, Inagaki L, et al. Lenvatinib for anaplastic thyroid cancer. *Front Oncol.* 2017;7:25. doi: [10.3389/fonc.2017.00025](https://doi.org/10.3389/fonc.2017.00025).

7. Higashiyama T, Sugino K, Hara H, Ito KI, Nakashima N, Onoda N, et al. Phase II study of the efficacy and safety of lenvatinib for anaplastic thyroid cancer (HOPE). *Eur J Cancer*. 2022;173:210–218. doi: [10.1016/j.ejca.2022.06.044](https://doi.org/10.1016/j.ejca.2022.06.044).

8. Nervo A, Gallo M, Samà MT, Felicetti F, Alfano M, Migliore E, et al. Lenvatinib in advanced radioiodine-refractory thyroid cancer: A snapshot of real-life clinical practice. *Anticancer Res.* 2018;38:1643–1649. doi: [10.21873/anticanres.12396](https://doi.org/10.21873/anticanres.12396).

9. Locati LD, Piovesan A, Durante C, Bregni M, Castagna MG, Zovato S, et al. Real-world efficacy and safety of lenvatinib: data from a compassionate use in the treatment of radioactive iodine-refractory differentiated thyroid cancer patients in Italy. *Eur J Cancer*. 2019;118:35–40. doi: [10.1016/j.ejca.2019.05.031](https://doi.org/10.1016/j.ejca.2019.05.031).

10. Jerkovich F, Califano I, Bueno F, Carrera JM, Giglio R, Abelleira E, et al. Real-life use of lenvatinib in patients with differentiated thyroid cancer: experience from Argentina. *Endocrine*. 2020;69:142–148. doi: [10.1007/s12020-020-02290-9](https://doi.org/10.1007/s12020-020-02290-9).

11. Rendl G, Sipos B, Becherer A, Sorko S, Trummer C, Raderer M, et al. Real-world data for lenvatinib in radioiodine-refractory differentiated thyroid cancer (RELEVANT): A retrospective multicentric analysis of clinical practice in Austria. *Int J Endocrinol.* 2020;2020:8834148. doi: [10.1155/2020/8834148](https://doi.org/10.1155/2020/8834148).

12. Porcelli T, Luongo C, Sessa F, Klain M, Masone S, Troncone G, et al. Long-term management of lenvatinib-treated thyroid cancer patients: a real-life experience at a single institution. *Endocrine*. 2021;73:358–366. doi: [10.1007/s12020-021-02634-z](https://doi.org/10.1007/s12020-021-02634-z).

13. Takahashi S, Kiyota N, Yamazaki T, Chayahara N, Nakano K, Inagaki L, et al. A Phase II study of the safety and efficacy of lenvatinib in patients with advanced thyroid cancer. *Future Oncol.* 2019;15:717–726. doi: [10.2217/fon-2018-0557](https://doi.org/10.2217/fon-2018-0557).

14. Yamazaki H, Iwasaki H, Takasaki H, Suganuma N, Sakai R, Masudo K, et al. Efficacy and tolerability of initial low-dose lenvatinib to treat differentiated thyroid cancer. *Medicine (Baltimore)*. 2019;98:e14774. doi: [10.1097/MD.0000000000014774](https://doi.org/10.1097/MD.0000000000014774).

15. Takahashi S, Tahara M, Ito K, Tori M, Kiyota N, Yoshida K, et al. Safety and effectiveness of lenvatinib in 594 patients with unresectable thyroid cancer in an All-Case post-marketing observational study in Japan. *Adv Ther.* 2020;37:3850–3862. doi: [10.1007/s12325-020-01433-8](https://doi.org/10.1007/s12325-020-01433-8).

16. Velcheti V, Hida T, Reckamp KL, Yang JC, Nokihara H, Sachdev P, et al. Phase 2 study of lenvatinib in patients with RET fusion-positive adenocarcinoma of the lung. *Eur J Cancer*. 2017;72:S178–S178. doi: [10.1016/S0959-8049(17)30651-2](https://doi.org/10.1016/S0959-8049(17)30651-2).

17. Sato J, Satouchi M, Itoh S, Okuma Y, Niho S, Mizugaki H, et al. Lenvatinib in patients with advanced or metastatic thymic carcinoma (remora): a multicentre, phase 2 trial. *Lancet Oncol.* 2020;21:843–850. doi: [10.1016/S1470-2045(20)30162-5](https://doi.org/10.1016/S1470-2045(20)30162-5).

18. Vergote I, Powell MA, Teneriello MG, Miller DS, Garcia AA, Mikheeva ON, et al. Second-line lenvatinib in patients with recurrent endometrial cancer. *Gynecol Oncol.* 2020;156:575–582. doi: [10.1016/j.ygyno.2019.12.039](https://doi.org/10.1016/j.ygyno.2019.12.039).

19. Capdevila J, Fazio N, Lopez C, Teulé A, Valle JW, Tafuto S, et al. Lenvatinib in patients with advanced Grade 1/2 pancreatic and gastrointestinal neuroendocrine tumors: results of the Phase II Talent trial (GETNE1509). *J Clin Oncol.* 2021;39:2304–2312. doi: [10.1200/JCO.20.03368](https://doi.org/10.1200/JCO.20.03368).

20. Iwasa S, Okita N, Kuchiba A, Ogawa G, Kawasaki M, Nakamura K, et al. Phase II study of lenvatinib for metastatic colorectal cancer refractory to standard chemotherapy: the LEMON study (NCCH1503). *ESMO Open*. 2020;5. doi: [10.1136/esmoopen-2020-000776](https://doi.org/10.1136/esmoopen-2020-000776).

21. Hamidi S, Boucher A, Lemieux B, Rondeau G, Lebœuf R, Ste-Marie LG, et al. Lenvatinib therapy for advanced thyroid cancer: real-life data on safety, efficacy, and some rare side effects. *J Endocr Soc.* 2022;6:bvac048. doi: [10.1210/jendso/bvac048](https://doi.org/10.1210/jendso/bvac048).

22. Kim SY, Kim SM, Chang H, Kim BW, Lee YS, Chang HS, et al. Safety of tyrosine kinase inhibitors in patients with differentiated thyroid cancer: real-world use of lenvatinib and sorafenib in Korea. *Front Endocrinol (Lausanne)*. 2019;10:384. doi: [10.3389/fendo.2019.00384](https://doi.org/10.3389/fendo.2019.00384).

23. Maesaka K, Sakamori R, Yamada R, Doi A, Tahata Y, Miyazaki M, et al. Comparison of atezolizumab plus bevacizumab and lenvatinib in terms of efficacy and safety as primary systemic chemotherapy for hepatocellular carcinoma. *Hepatol Res.* 2022;52:630–640. doi: [10.1111/hepr.13771](https://doi.org/10.1111/hepr.13771).

24. Kim BK, Cheon J, Kim H, Kang B, Ha Y, Kim DY, et al. Atezolizumab/Bevacizumab vs. lenvatinib as First-Line Therapy for unresectable Hepatocellular Carcinoma: A Real-World, Multi-Center Study. *Cancers (Basel)*. 2022;14. doi: [10.3390/cancers14071747](https://doi.org/10.3390/cancers14071747).

25. Hiraoka A, Kumada T, Tada T, Hirooka M, Kariyama K, Tani J, et al. Does first-line treatment have prognostic impact for unresectable HCC?-Atezolizumab plus bevacizumab versus lenvatinib. *Cancer Med.* 2023;12:325–334. doi: [10.1002/cam4.4854](https://doi.org/10.1002/cam4.4854).

26. Niizeki T, Tokunaga T, Takami Y, Wada Y, Harada M, Shibata M, et al. Comparison of efficacy and safety of atezolizumab plus bevacizumab and lenvatinib as first-line therapy for unresectable hepatocellular carcinoma: A propensity score matching analysis. *Target Oncol.* 2022;17:643–653. doi: [10.1007/s11523-022-00921-x](https://doi.org/10.1007/s11523-022-00921-x).

27. Su CW, Teng W, Lin PT, Jeng WJ, Chen KA, Hsieh YC, et al. Similar efficacy and safety between lenvatinib versus atezolizumab plus bevacizumab as the first-line treatment for unresectable hepatocellular carcinoma. *Cancer Med.* 2023;12:7077–7089. doi: [10.1002/cam4.5506](https://doi.org/10.1002/cam4.5506).

28. Casadei-Gardini A, Rimini M, Tada T, Suda G, Shimose S, Kudo M, et al. Atezolizumab plus bevacizumab versus lenvatinib for unresectable hepatocellular carcinoma: a large real-life worldwide population. *Eur J Cancer*. 2023;180:9–20. doi: [10.1016/j.ejca.2022.11.017](https://doi.org/10.1016/j.ejca.2022.11.017).

29. Tada T, Kumada T, Hiraoka A, Michitaka K, Atsukawa M, Hirooka M, et al. Safety and efficacy of lenvatinib in elderly patients with unresectable hepatocellular carcinoma: A multicenter analysis with propensity score matching. *Hepatol Res.* 2020;50:75–83. doi: [10.1111/hepr.13427](https://doi.org/10.1111/hepr.13427).

30. Wei F, Huang Q, He J, Luo L, Zeng Y. Lenvatinib Plus Camrelizumab versus Lenvatinib monotherapy as Post-Progression Treatment for Advanced Hepatocellular Carcinoma: A Short-Term Prognostic Study. *Cancer Manag Res.* 2021;13:4233–4240. doi: [10.2147/CMAR.S304820](https://doi.org/10.2147/CMAR.S304820).

31. Chen K, Wei W, Liu L, Deng ZJ, Li L, Liang XM, et al. Lenvatinib with or without immune checkpoint inhibitors for patients with unresectable hepatocellular carcinoma in real-world clinical practice. *Cancer Immunol Immunother.* 2022;71:1063–1074. doi: [10.1007/s00262-021-03060-w](https://doi.org/10.1007/s00262-021-03060-w).

32. Zhu Y, Sun P, Wang K, Xiao S, Cheng Y, Li X, et al. Efficacy and safety of lenvatinib monotreatment and lenvatinib-based combination therapy for patients with unresectable hepatocellular carcinoma: a retrospective, real-world study in China. *Cancer Cell Int.* 2021;21:503. doi: [10.1186/s12935-021-02200-7](https://doi.org/10.1186/s12935-021-02200-7).

33. Li Q, Cao M, Yuan G, Cheng X, Zang M, Chen M, et al. Lenvatinib Plus Camrelizumab vs. Lenvatinib monotherapy as First-Line Treatment for unresectable Hepatocellular Carcinoma: A Multicenter Retrospective Cohort Study. *Front Oncol.* 2022;12:809709. doi: [10.3389/fonc.2022.809709](https://doi.org/10.3389/fonc.2022.809709).

34. Zhao L, Chang N, Shi L, Li F, Meng F, Xie X, et al. Lenvatinib plus sintilimab versus lenvatinib monotherapy as first-line treatment for advanced HBV-related hepatocellular carcinoma: A retrospective, real-world study. *Heliyon*. 2022;8:e09538. doi: [10.1016/j.heliyon.2022.e09538](https://doi.org/10.1016/j.heliyon.2022.e09538).

35. He MK, Liang RB, Zhao Y, Xu YJ, Chen HW, Zhou YM, et al. Lenvatinib, toripalimab, plus hepatic arterial infusion chemotherapy versus lenvatinib alone for advanced hepatocellular carcinoma. *Ther Adv Med Oncol.* 2021;13:17588359211002720. doi: [10.1177/17588359211002720](https://doi.org/10.1177/17588359211002720).

36. Kudo M, Finn RS, Qin S, Han KH, Ikeda K, Piscaglia F, et al. Lenvatinib versus sorafenib in first-line treatment of patients with unresectable hepatocellular carcinoma: a randomised phase 3 non-inferiority trial. *Lancet*. 2018;391:1163–1173. doi: [10.1016/S0140-6736(18)30207-1](https://doi.org/10.1016/S0140-6736(18)30207-1).

37. Rimini M, Shimose S, Lonardi S, Tada T, Masi G, Iwamoto H, et al. Lenvatinib versus sorafenib as first-line treatment in hepatocellular carcinoma: A multi-institutional matched case-control study. *Hepatol Res.* 2021;51:1229–1241. doi: [10.1111/hepr.13718](https://doi.org/10.1111/hepr.13718).

38. Lee J, Sung PS, Yang H, Lee SK, Nam HC, Yoo SH, et al. A real-world comparative analysis of lenvatinib and sorafenib as a salvage therapy for transarterial treatments in unresectable HCC. *J Clin Med.* 2020;9. doi: [10.3390/jcm9124121](https://doi.org/10.3390/jcm9124121).

39. Park MK, Lee YB, Moon H, Choi NR, Kim MA, Jang H, et al. Effectiveness of lenvatinib versus sorafenib for unresectable hepatocellular carcinoma in patients with hepatic decompensation. *Dig Dis Sci.* 2022;67:4939–4949. doi: [10.1007/s10620-021-07365-9](https://doi.org/10.1007/s10620-021-07365-9).

40. Marrocco GA, Silletta M, Bianco V, Mondera F, Sciortino C, Pappalardo L, et al. Lenvatinib versus sorafenib in advanced hepatic cell carcinoma: A double center retrospective analysis. *Anticancer Res.* 2023;43:755–763. doi: [10.21873/anticanres.16215](https://doi.org/10.21873/anticanres.16215).

41. Hata K, Suetsugu K, Egashira N, Makihara Y, Itoh S, Yoshizumi T, et al. Association of lenvatinib plasma concentration with clinical efficacy and adverse events in patients with hepatocellular carcinoma. *Cancer Chemother Pharmacol.* 2020;86:803–813. doi: [10.1007/s00280-020-04178-x](https://doi.org/10.1007/s00280-020-04178-x).

42. Kobayashi S, Fukushima T, Ueno M, Moriya S, Chuma M, Numata K, et al. A prospective observational cohort study of lenvatinib as initial treatment in patients with BCLC-defined stage B hepatocellular carcinoma. *BMC Cancer*. 2022;22:517. doi: [10.1186/s12885-022-09625-x](https://doi.org/10.1186/s12885-022-09625-x).

43. Ikeda K, Kudo M, Kawazoe S, Osaki Y, Ikeda M, Okusaka T, et al. Phase 2 study of lenvatinib in patients with advanced hepatocellular carcinoma. *J Gastroenterol.* 2017;52:512–519. doi: [10.1007/s00535-016-1263-4](https://doi.org/10.1007/s00535-016-1263-4).

44. Tokunaga T, Tateyama M, Tanaka K, Narahara S, Inada H, Kurano S, et al. Optimal management of lenvatinib therapy for patients with unresectable hepatocellular carcinoma by balancing the therapeutic effect with the relative dose intensity. *Hepatol Res.* 2022;52:105–119. doi: [10.1111/hepr.13720](https://doi.org/10.1111/hepr.13720).

45. Hiraoka A, Kumada T, Kariyama K, Takaguchi K, Itobayashi E, Shimada N, et al. Therapeutic potential of lenvatinib for unresectable hepatocellular carcinoma in clinical practice: multicenter analysis. *Hepatol Res.* 2019;49:111–117. doi: [10.1111/hepr.13243](https://doi.org/10.1111/hepr.13243).

46. Hiraoka A, Kumada T, Kariyama K, Takaguchi K, Atsukawa M, Itobayashi E, et al. Clinical features of lenvatinib for unresectable hepatocellular carcinoma in real-world conditions: multicenter analysis. *Cancer Med.* 2019;8:137–146. doi: [10.1002/cam4.1909](https://doi.org/10.1002/cam4.1909).

47. Obi S, Sato T, Sato S, Kanda M, Tokudome Y, Kojima Y, et al. The efficacy and safety of lenvatinib for advanced hepatocellular carcinoma in a real-world setting. *Hepatol Int.* 2019;13:199–204. doi: [10.1007/s12072-019-09929-4](https://doi.org/10.1007/s12072-019-09929-4).

48. Hatanaka T, Kakizaki S, Nagashima T, Namikawa M, Tojima H, Shimada Y, et al. Analyses of objective response rate, progression-free survival, and adverse events in hepatocellular carcinoma patients treated with lenvatinib: A multicenter retrospective study. *Hepatol Res.* 2020;50:382–395. doi: [10.1111/hepr.13460](https://doi.org/10.1111/hepr.13460).

49. Ohki T, Sato K, Kondo M, Goto E, Sato T, Kondo Y, et al. Impact of adverse events on the progression-free survival of patients with advanced hepatocellular carcinoma treated with lenvatinib: A multicenter retrospective study. *Drugs Real World Outcomes*. 2020;7:141–149. doi: [10.1007/s40801-020-00179-7](https://doi.org/10.1007/s40801-020-00179-7).

50. Sho T, Suda G, Ogawa K, Kimura M, Shimazaki T, Maehara O, et al. Early response and safety of lenvatinib for patients with advanced hepatocellular carcinoma in a real-world setting. *JGH Open*. 2020;4:54–60. doi: [10.1002/jgh3.12209](https://doi.org/10.1002/jgh3.12209).

51. Fuchigami A, Imai Y, Uchida Y, Uchiya H, Fujii Y, Nakazawa M, et al. Therapeutic efficacy of lenvatinib for patients with unresectable hepatocellular carcinoma based on the middle-term outcome. *PLoS ONE*. 2020;15:e0231427. doi: [10.1371/journal.pone.0231427](https://doi.org/10.1371/journal.pone.0231427).

52. Sho T, Suda G, Ogawa K, Shigesawa T, Suzuki K, Nakamura A, et al. Lenvatinib in patients with unresectable hepatocellular carcinoma who do not meet the REFLECT trial eligibility criteria. *Hepatol Res.* 2020;50:966–977. doi: [10.1111/hepr.13511](https://doi.org/10.1111/hepr.13511).

53. Wang DX, Yang X, Lin JZ, Bai Y, Long JY, Yang XB, et al. Efficacy and safety of lenvatinib for patients with advanced hepatocellular carcinoma: A retrospective, real-world study conducted in China. *World J Gastroenterol.* 2020;26:4465–4478. doi: [10.3748/wjg.v26.i30.4465](https://doi.org/10.3748/wjg.v26.i30.4465).

54. Cheon J, Chon HJ, Bang Y, Park NH, Shin JW, Kim KM, et al. Real-world efficacy and safety of lenvatinib in Korean patients with advanced hepatocellular carcinoma: A multicenter retrospective analysis. *Liver Cancer*. 2020;9:613–624. doi: [10.1159/000508901](https://doi.org/10.1159/000508901).

55. Aoki T, Kudo M, Ueshima K, Morita M, Chishina H, Takita M, et al. Exploratory analysis of lenvatinib therapy in patients with unresectable hepatocellular carcinoma who have failed prior PD-1/PD-L1 checkpoint blockade. *Cancers (Basel)*. 2020;12. doi: [10.3390/cancers12103048](https://doi.org/10.3390/cancers12103048).

56. Han B, Ding H, Zhao S, Zhang Y, Wang J, Zhang Y, et al. Potential role of adjuvant lenvatinib in improving disease-free survival for patients with high-risk hepatitis B virus-related hepatocellular carcinoma following liver transplantation: A retrospective, case control study. *Front Oncol.* 2020;10:562103. doi: [10.3389/fonc.2020.562103](https://doi.org/10.3389/fonc.2020.562103).

57. Goh MJ, Oh JH, Park Y, Kim J, Kang W, Sinn DH, et al. Efficacy and safety of lenvatinib therapy for unresectable hepatocellular carcinoma in a real-world practice in Korea. *Liver Cancer*. 2021;10:52–62. doi: [10.1159/000512239](https://doi.org/10.1159/000512239).

58. Sasaki R, Fukushima M, Haraguchi M, Miuma S, Miyaaki H, Hidaka M, et al. Liver function in older patients with unresectable hepatocellular carcinoma after administration of lenvatinib. *Anticancer Res.* 2021;41:2025–2032. doi: [10.21873/anticanres.14970](https://doi.org/10.21873/anticanres.14970).

59. Tomonari T, Sato Y, Tanaka H, Mitsuhashi T, Hirao A, Tanaka T, et al. Therapeutic efficacy of lenvatinib in nonviral unresectable hepatocellular carcinoma. *JGH Open*. 2021;5:1275–1283. doi: [10.1002/jgh3.12663](https://doi.org/10.1002/jgh3.12663).

60. Shimozato N, Namisaki T, Okano A, Ohana M, Kinoshita D, Kawasaki T, et al. Efficacy and safety of lenvatinib for patients with advanced hepatocellular carcinoma: A retrospective, real-world study conducted in Japan. *Anticancer Res.* 2022;42:173–183. doi: [10.21873/anticanres.15471](https://doi.org/10.21873/anticanres.15471).

61. Casadei-Gardini A, Rimini M, Kudo M, Shimose S, Tada T, Suda G, et al. Real life study of lenvatinib therapy for hepatocellular carcinoma: RELEVANT study. *Liver Cancer*. 2022;11:527–539. doi: [10.1159/000525145](https://doi.org/10.1159/000525145).

62. Furuse J, Izumi N, Motomura K, Inaba Y, Katamura Y, Kondo Y, et al. Safety and effectiveness of lenvatinib in patients with unresectable hepatocellular carcinoma in real-world clinical practice: an observational post-marketing study in Japan. *Drugs Real World Outcomes*. 2023;10:195–205. doi: [10.1007/s40801-022-00348-w](https://doi.org/10.1007/s40801-022-00348-w).

63. Chen YH, Chen YY, Wang JH, Hung CH. Efficacy and safety of lenvatinib after progression on first-line atezolizumab plus bevacizumab treatment in advanced hepatocellular carcinoma patients. *Anticancer Res.* 2023;43:1377–1384. doi: [10.21873/anticanres.16286](https://doi.org/10.21873/anticanres.16286).

64. Wang Y, Yang X, Wang D, Yang X, Wang Y, Long J, et al. Lenvatinib beyond first-line therapy in patients with advanced biliary tract carcinoma. *Front Oncol.* 2022;12:785535. doi: [10.3389/fonc.2022.785535](https://doi.org/10.3389/fonc.2022.785535).
